# Research Opportunities in Autonomic Neural Mechanisms of Cardiopulmonary Regulation: A Report From the National Heart, Lung, and Blood Institute and the National Institutes of Health Office of the Director Workshop

**DOI:** 10.1016/j.jacbts.2021.11.003

**Published:** 2022-01-26

**Authors:** Reena Mehra, Olga A. Tjurmina, Olujimi A. Ajijola, Rishi Arora, Donald C. Bolser, Mark W. Chapleau, Peng-Sheng Chen, Colleen E. Clancy, Brian P. Delisle, Michael R. Gold, Jeffrey J. Goldberger, David S. Goldstein, Beth A. Habecker, M. Louis Handoko, Robert Harvey, James P. Hummel, Thomas Hund, Christian Meyer, Susan Redline, Crystal M. Ripplinger, Marc A. Simon, Virend K. Somers, Stavros Stavrakis, Thomas Taylor-Clark, Bradley Joel Undem, Richard L. Verrier, Irving H. Zucker, George Sopko, Kalyanam Shivkumar

**Affiliations:** a Cleveland Clinic, Cleveland, Ohio, USA; b Case Western Reserve University, Cleveland, Ohio, USA; c National Heart, Lung, and Blood Institute, Bethesda, Maryland, USA; d David Geffen School of Medicine at UCLA, Los Angeles, California, USA; e Feinberg School of Medicine at Northwestern University, Chicago, Illinois, USA; f University of Florida, Gainesville, Florida, USA; g University of Iowa Carver College of Medicine, Iowa City, Iowa, USA; h Cedars-Sinai Medical Center, Los Angeles, California, USA; i University of California Davis, Davis, California, USA; j University of Kentucky, Lexington, Kentucky, USA; k Medical University of South Carolina, Charleston, South Carolina, USA; l University of Miami Miller School of Medicine, Miami, Florida, USA; m National Institute of Neurological Disorders and Stroke, Bethesda, Maryland, USA; n Oregon Health and Science University School of Medicine, Portland, Oregon, USA; o Amsterdam University Medical Centers, Amsterdam, the Netherlands; p University of Nevada Reno, Reno, Nevada, USA; q Yale University School of Medicine, New Haven, Connecticut, USA; r Ohio State University, Columbus, Ohio, USA; s University of Düsseldorf, Germany, Düsseldorf, Germany; t Harvard Medical School, Boston, Massachusetts, USA; u University of California Davis School of Medicine, Davis, California, USA; v University of Pittsburgh Medical Center, Pittsburgh, Pennsylvania, USA; w University of California-San Francisco, San Francisco, California, USA; x Mayo Clinic, Rochester, Minnesota, USA; y University of Oklahoma Health Sciences Center, Oklahoma City, Oklahoma, USA; z University of South Florida, Tampa, Florida, USA; aa Johns Hopkins University, Baltimore, Maryland, USA; bb Beth Israel Deaconess Medical Center, Harvard Medical School, Boston, Massachusetts, USA; cc University of Nebraska Medical Center, Omaha, Nebraska, USA

**Keywords:** asthma, atrial fibrillation, autonomic nervous system, cardiopulmonary, chronic obstructive pulmonary disease, circadian, heart failure, pulmonary arterial hypertension, sleep apnea, ventricular arrhythmia, ACE, angiotensin-converting enzyme, Ach, acetylcholine, AD, autonomic dysregulation, AF, atrial fibrillation, ANS, autonomic nervous system, CNS, central nervous system, COPD, chronic obstructive pulmonary disease, CSA, central sleep apnea, CVD, cardiovascular disease, ECG, electrocardiogram, EV, extracellular vesicle, GP, ganglionated plexi, HF, heart failure, HFrEF, heart failure with reduced ejection fraction, HFpEF, heart failure with preserved ejection fraction, HRV, heart rate variability, LQT, long QT, MI, myocardial infarction, NE, norepinephrine, NHLBI, National Heart, Lung, and Blood Institute, NPY, neuropeptide Y, NREM, non-rapid eye movement, OSA, obstructive sleep apnea, PAH, pulmonary arterial hypertension, PV, pulmonary vein, REM, rapid eye movement, RV, right ventricular, SCD, sudden cardiac death, SDB, sleep disordered breathing, SNA, sympathetic nerve activity, SNSA, sympathetic nervous system activity, TLD, targeted lung denervation

## Abstract

This virtual workshop was convened by the National Heart, Lung, and Blood Institute, in partnership with the Office of Strategic Coordination of the Office of the National Institutes of Health Director, and held September 2 to 3, 2020. The intent was to assemble a multidisciplinary group of experts in basic, translational, and clinical research in neuroscience and cardiopulmonary disorders to identify knowledge gaps, guide future research efforts, and foster multidisciplinary collaborations pertaining to autonomic neural mechanisms of cardiopulmonary regulation. The group critically evaluated the current state of knowledge of the roles that the autonomic nervous system plays in regulation of cardiopulmonary function in health and in pathophysiology of arrhythmias, heart failure, sleep and circadian dysfunction, and breathing disorders. Opportunities to leverage the Common Fund’s SPARC (Stimulating Peripheral Activity to Relieve Conditions) program were characterized as related to nonpharmacologic neuromodulation and device-based therapies. Common themes discussed include knowledge gaps, research priorities, and approaches to develop novel predictive markers of autonomic dysfunction. Approaches to precisely target neural pathophysiological mechanisms to herald new therapies for arrhythmias, heart failure, sleep and circadian rhythm physiology, and breathing disorders were also detailed.

## Highlights

• The ANS is a key regulator of cardiopulmonary health and disease and sleep/circadian pathophysiology.
• Understanding cardiopulmonary sympathetic and parasympathetic nerve structure/function over disease time-course and cell type interactions is essential.
• In vitro autonomic nervous system and experimental and computational integrative studies are necessary.
• Clarifying sex-and race-specific cardiopulmonary and sleep/circadian influence on autonomic nerve function in response to neurotherapeutic interventions is critical to inform personalized strategies.

Advances in autonomic nervous system (ANS) research in recent years have led to numerous discoveries that have initiated a return of this classic discipline back to the center stage of health and disease. At the same time, a number of challenges and knowledge gaps have been recognized to clarify the role of the ANS in cardiopulmonary diseases and to leverage ANS science for human therapy. As such, the National Heart, Lung, and Blood Institute (NHLBI), in partnership with the Office of Strategic Coordination of the Office of the National Institutes of Health Director, convened a workshop on “Autonomic Neural Mechanisms of Cardiopulmonary Regulation” September 2 to 3, 2020. The workshop executive summary and conference recording can be found at NHLBI Events. The workshop brought together a multidisciplinary and international group of experts in basic, translational, and clinical research in neuroscience and cardiopulmonary disorders, and more than 200 representatives of multiple federal and nonfederal agencies and academic institutions.

The premise of the workshop was driven by a need to articulate gaps and research priorities specific to the ANS—the latter responsible for regulation of cardiac, vascular, and pulmonary physiology via maintaining a balance of sympathetic and parasympathetic outputs to the heart, vasculature, and lungs in response to stimuli. The ANS plays a key role in the development and progression of cardiopulmonary disease and in sleep and circadian rhythm pathophysiology. To this end, ANS-targeted interventions have been developed to target ANS impairment (1). This includes pharmacologic therapies directed toward modulation of sympathetic nervous system activation such as beta-blockers, while recognizing limitations of off-target action and long-term side effects. Nonpharmacologic interventions such as vagal nerve stimulation, renal and carotid body denervation, and stellectomy are other options; however, they are invasive, expensive, and also associated with side effects (2). Furthermore, there are limitations with human model systems including heart rate, heart rate variability (HRV), plasma catecholamines, and noradrenaline spillover that can have limitations in terms of largescale implementation, can be confounded in certain states such as heart failure (HF), and have age- and sex-specific influences that need to be considered (3). The overarching conceptual framework of opportunities presented in this report is characterized by a better understanding of the complex, hierarchical neural networks in health and cardiopulmonary disease to develop more sophisticated tools and interventions to inform ANS research discovery and interventional studies (4, 5, 6) (Central Illustration).

**Central Illustration:**
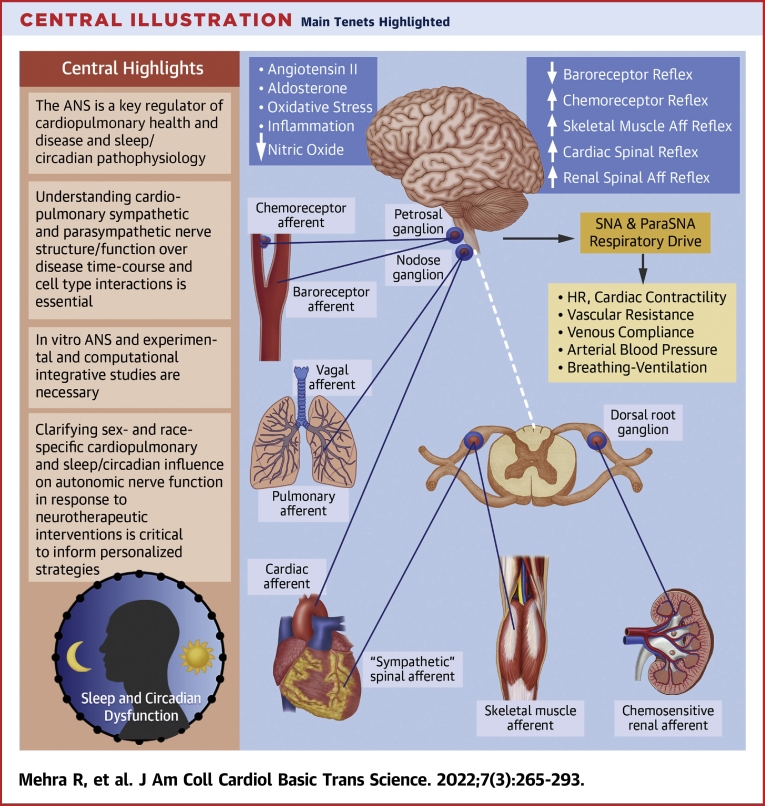
Main Tenets Highlighted The premise of the workshop is based upon a need to articulate gaps and research priorities specific to the ANS—responsible for regulation of cardiac, vascular, and pulmonary physiology via maintaining a balance of sympathetic and parasympathetic outputs to the heart, vasculature, and lungs in response to stimuli. The ANS plays a key role in the development and progression of cardiopulmonary disease and in sleep and circadian rhythm pathophysiology. Aff = afferent; ANS = autonomic nervous system; DRG = dorsal root ganglia; HR = heart rate; ParaSNA = parasympathetic nerve activity; SNA = sympathetic nerve activity.

Therefore, the workshop was structured to address 6 topic areas: 1) fundamental mechanisms of neural signaling in development and progression of cardiopulmonary diseases; 2) ANS-related pathophysiology of HF; 3) neuroelectrophysiological contributions to atrial arrhythmias; 4) neuroelectrophysiological aspects of ventricular arrhythmia; 5) ANS alterations in cardiopulmonary-related sleep and circadian disorders; and 6) neurophysiological mechanisms in pulmonary diseases and interactions with cardiac function. Although this report is topically organized, we appreciate the synergies and intertwining biological aspects of these key areas. The overarching impetus was to identify the highest priority research gaps and opportunities to accelerate key discoveries in neural science and neurotherapeutics in cardiopulmonary and sleep and circadian rhythm disorders. Importantly, targeting the ANS is a major therapeutic opportunity, and therefore, key to this is understanding the neurobiology of end-organ function in normal and disease states. This report presents a summary of cross-cutting themes, challenges, opportunities, and available resources discussed at the workshop.

## Fundamental Mechanisms of Neural Signaling in Development and Progression of Cardiopulmonary Diseases

### Background

The ANS plays a key role in the regulation of cardiac, vascular, and pulmonary function. Sensory afferent fibers innervating the heart, vasculature, and lungs include mechanosensitive nerves that detect changes in cardiac stretch, lung stretch, blood pressure, and blood volume, and chemosensitive fibers that are normally quiescent, but are activated by stimuli such as ischemia and inflammation (7). Information transmitted to the brainstem (8,9) via dorsal root ganglia or the vagus nerve (with distinct nodose and jugular neural pathways) is integrated with information from higher brain centers to regulate cardiopulmonary control through activation or inhibition of parasympathetic and sympathetic outflow. Parasympathetic nerves projecting to the heart and lungs are predominantly cholinergic and have negative chronotropic, dromotropic, and inotropic effects while stimulating bronchoconstriction and mucus secretion in the airways. Sympathetic nerves innervating those targets are noradrenergic and contain neuropeptide Y (NPY) (10) and other cotransmitters/neuropeptides. Release of norepinephrine (NE) has positive chronotropic, dromotropic, and inotropic effects (11), but sympathetic activation has a limited effect on airway smooth muscle or mucus secretion (although circulating epinephrine causes bronchodilation). Parasympathetic transmission predominates in healthy humans, and decreased cardiac parasympathetic tone or the ratio of sympathetic to parasympathetic tone are powerful predictors of susceptibility to arrhythmia and death.

**Figure 1:**
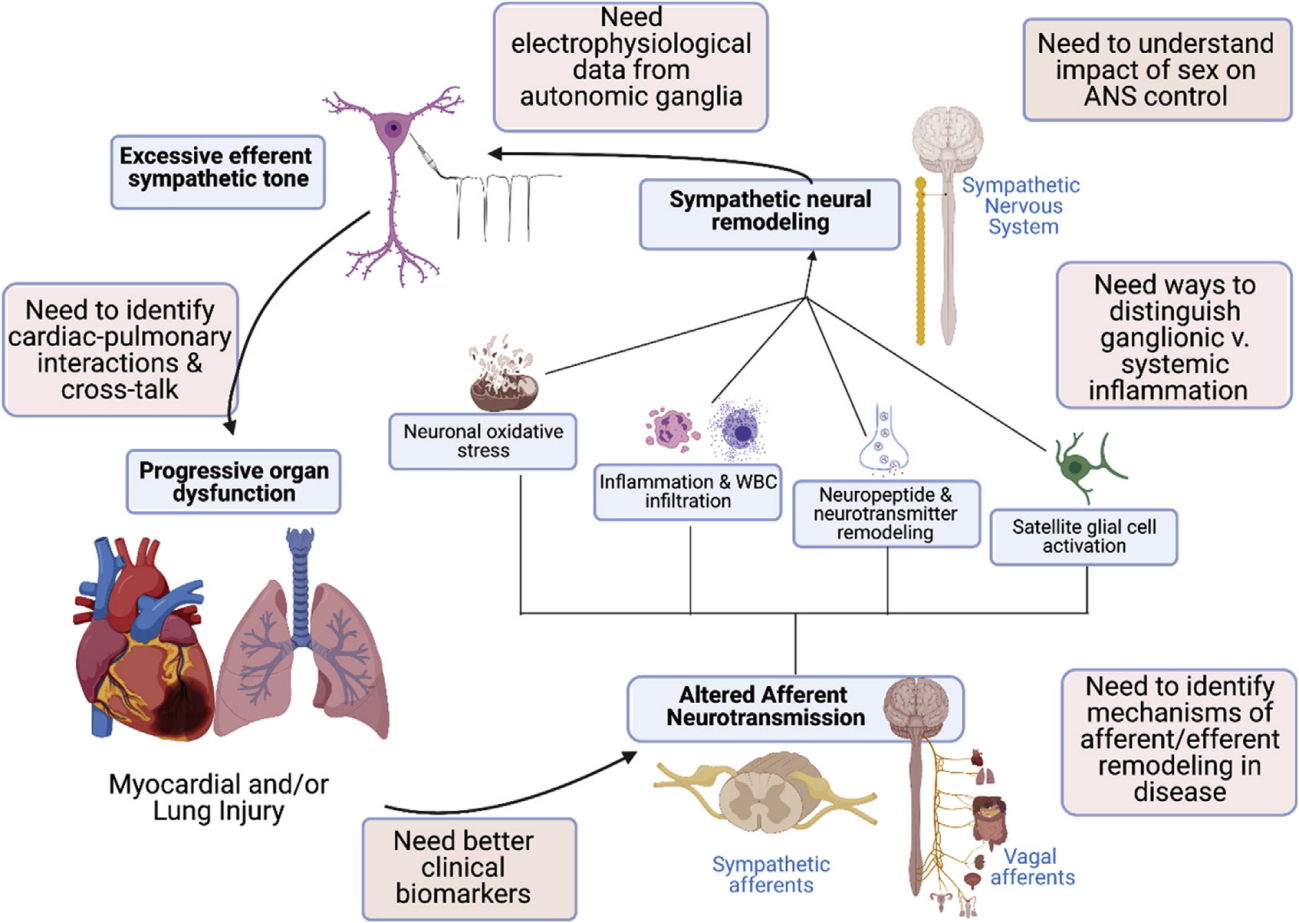
Fundamental Mechanisms of Neural Signaling in Development and Progression of Cardiopulmonary Disease Normal cardiopulmonary reflexes are disrupted in disease, leading to increased sympathetic and decreased parasympathetic transmission. Injury activates afferent nerves that mediate sympathoexcitatory-positive feedback reflexes that contribute to myocardial and/or lung injury. We do not adequately understand **(clockwise from top)** the electrophysiological and biophysical properties of autonomic ganglia; the impact of sex as a biological variable; how to distinguish the roles of ganglionic versus systemic inflammation in neural remodeling; the mechanisms that drive afferent and efferent remodeling during disease; how to integrate clinical data from a variety of sources, scales, and modalities to guide therapy for specific patients; and the nature of interactions between cardiac and pulmonary nerves.

Normal cardiopulmonary reflexes are disrupted in disease states, for example, ischemia, hypertension, and chronic HF, leading to increased sympathetic and decreased parasympathetic transmission (Figure 1). In some instances, ischemia or mechanical changes activate afferents that mediate sympathoexcitatory, positive feedback reflexes in a nonhomeostatic way (12,13). Excessive sympathetic transmission contributes to cardiac hypertrophy and fibrosis, and can trigger arrhythmias (14). This pathologic increase in sympathetic outflow can be blunted by selective destruction of cardiac spinal afferents using resiniferatoxin (14,15). Cardiopulmonary and metabolic diseases also lead to decreased efferent parasympathetic transmission, which increases arrhythmia risk. Unfortunately, we do not fully understand cellular electrophysiological function within peripheral ganglia or the associated brainstem circuits (16), nor the mechanisms that cause ganglionic remodeling and contribute to aberrant reflexes in disease, nor the impact of sex as a biological variable on these processes. Crosstalk between cardiopulmonary afferent stimulation and sympathetic modulation of visceral organs is likewise poorly understood. For example, inhalation of the irritant allyl isothiocyanate (AITC) stimulates vagal afferent sensory nerves to trigger reflex bradycardia in awake healthy animals (17), but in hypertensive animals, AITC evokes reflex tachyarrhythmia (18). New approaches are needed to characterize the distinct types of afferent and efferent nerves that innervate the cardiopulmonary system and determine how they interact with one another and remodel in disease states.

Peripheral neuroinflammation occurs in many diseases that cause autonomic imbalance, including myocardial infarction (MI) and HF (19, 20, 21). The precise inciting factors, timeline of the inflammatory stimulus, the cell types involved, and the critical role played by inflammation in promoting autonomic and cardiopulmonary dysfunction are poorly understood. Lung inflammation is a characteristic of many diseases, and various signaling pathways have been implicated in dysfunction and remodeling of afferent and autonomic signaling, although there are significant gaps in our mechanistic understanding. The inflammatory response that occurs in the heart after ischemia-reperfusion injury has been characterized in detail, but MI also leads to accumulation of immune cells in stellate ganglia (sympathetic), intrinsic cardiac ganglia (primarily parasympathetic), and dorsal root ganglia (sensory). Activation of satellite glial cells and neuronal oxidative stress in stellate ganglia are also features of chronic cardiac disease. We have a limited understanding of the triggers for inflammation, glial activation, and oxidative stress within peripheral ganglia during cardiopulmonary disease. Distinguishing altered neurotransmission driven by systemic inflammation (ie, elevated circulating proinflammatory cytokines) from local intraganglionic inflammation presents a challenge. There may be therapeutic potential in targeting local intraganglionic inflammation to mitigate or treat the excess sympathetic and impaired parasympathetic transmission that contributes to cardiopulmonary pathology. Conversely, autonomic transmission can modulate inflammatory responses (22), and there may be therapeutic potential in targeting nerves to alter the immune response in cardiopulmonary disease.

In addition to hard-wired neural pathways between cardiopulmonary regions and the brain, there are novel non-neural communication pathways that can modulate autonomic tone. Extracellular vesicles (EVs) are a prime example of such communication (23). Transfer of proteins, lipids, nucleotides, and metabolites from one organ to another is mediated by EV cargo, specific to each tissue. These cargoes may play an important role in crosstalk between visceral organs and the ANS, because they modify the physiology of other neuronal circuits (24). The use of EVs as delivery vehicles for therapeutic purposes is growing, with the heart an early target of choice (25). The tools now exist to support identification of multiple EV cargoes (24), opening the way for new investigation into the role of EVs in autonomic regulatory mechanisms in health and disease.

Genetic models have become more valuable as tools to investigate cardiopulmonary regulation. The advent of approaches such as Cre/lox and Flp/FRT recombination that allow deletion or expression of genes in a cell-specific manner facilitate in vivo experiments. Inducible recombination with CreERT2 or the Tet On/Off system that allows for induction and then suppression of a gene further refine the strategies that can be used to elucidate mechanisms of disease. Genetic models have been valuable in controlling neurotransmission with optogenetic or chemogenetic approaches, and facilitating characterization of genetically distinct afferents and efferents innervating the cardiopulmonary system. As more neuronal subtypes are identified, these methods, combined with additional technologies such as single-cell RNA sequencing and tissue clearing, will allow us to determine the cellular basis for ganglionic remodeling and baroreflex dysfunction in HF and other diseases. These approaches also show promise for dissociating the impact of peripheral versus systemic inflammation on cardiopulmonary homeostasis, identifying potential sex differences, and may facilitate development of new biomarkers and therapeutic interventions.

### Knowledge gaps

1. Limited data are available describing cellular electrophysiology and biophysics within intact peripheral ganglia, or the mechanisms of afferent, efferent, and reflex remodeling in cardiovascular and respiratory diseases.
2. The factors inciting intraganglionic inflammation are not understood, nor are the cellular interactions driven by inflammation (microglia ↔ satellite glia ↔ T cells ↔ neurons) at the systemic and intraganglionic levels.
3. Non-neural modes of sympathetic modulation (eg, extracellular vesicular cargo) in normal and stressed states are not clearly understood.
4. There is a need to characterize genetically/functionally different afferent and efferent nerves that innervate the cardiopulmonary system, including their anatomical distribution, identification of biomarkers, and incorporation of clinical modalities and therapy.
5. Reflex interactions and organ crosstalk between cardiac and pulmonary afferent stimulation need to be identified and differentiated in male and female animals.

### Research priorities

1. Understand integrative reflex control of the cardiopulmonary system in health and disease, including cellular diversity, neurochemistry, electrophysiology, and the mechanisms of afferent, efferent, and reflex remodeling in males and females.
2. Understand the role of neuroinflammation in cardiopulmonary disease progression, including identifying key cell types involved in intraganglionic inflammation; characterizing the actions of pro- and anti-inflammatory cytokines on electrophysiological and neurochemical remodeling of neurons and glia; and assessing the therapeutic potential of targeting inflammation to treat cardiopulmonary disorders.
3. Develop and use sophisticated modeling and simulations of the cardiopulmonary neural hierarchy to reveal mechanisms of autonomic dysfunction in disease progression.

## ANS-Related Pathophysiology of HF

### Background

#### Neural remodeling in HF

**Figure 2:**
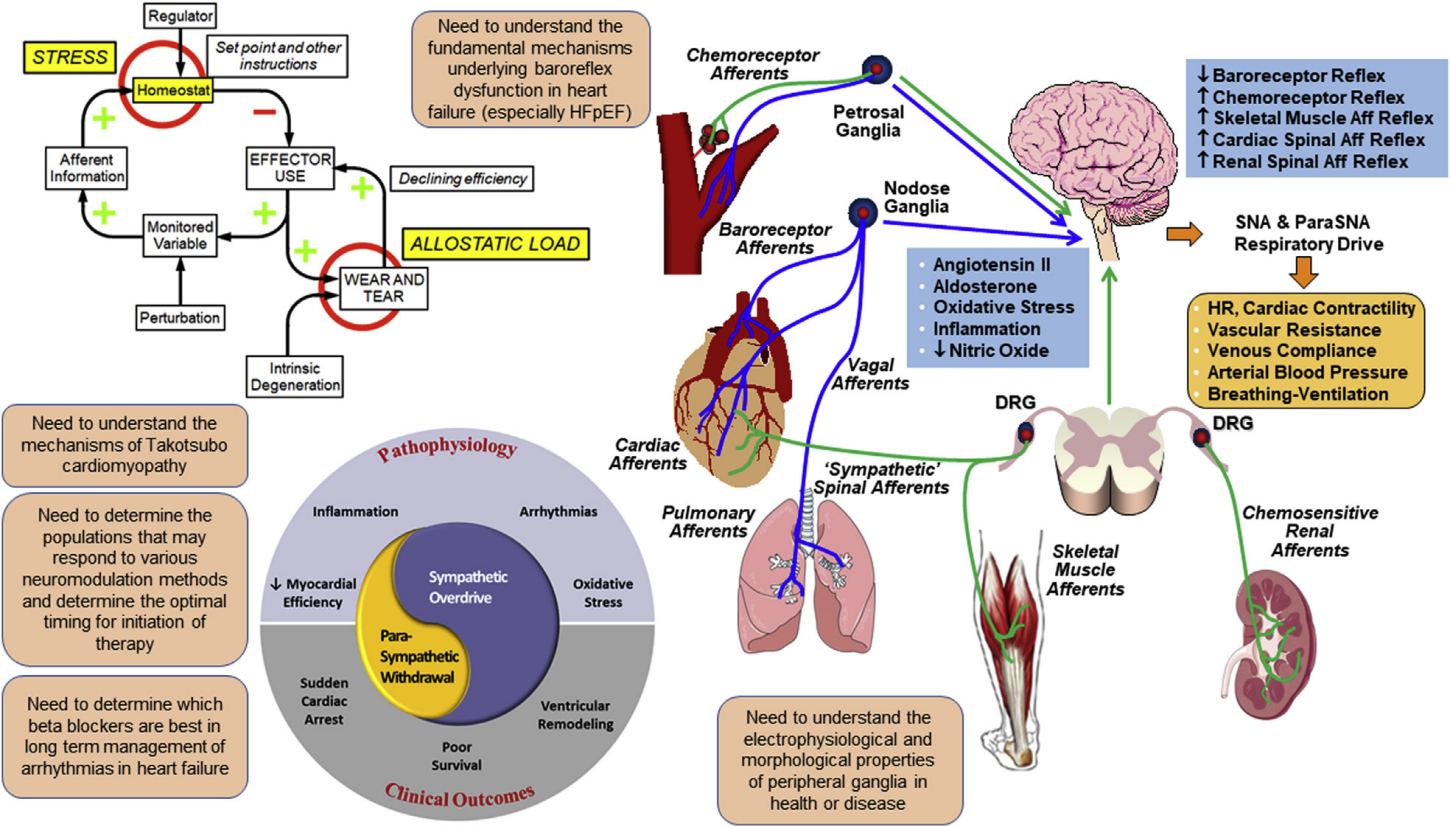
ANS-Related Pathophysiology of HF Heart failure (HF) is associated with significant neural remodeling characterized by increased sympathetic and reduced parasympathetic nerve activity. The **central panel** shows that the autonomic nervous system (ANS) remodeling contributes to the pathophysiology of heart failure and affects the clinical outcomes. The **left panel**, adapted from Goldstein (201) shows a concept diagram relating stress to chronic disorders such as heart failure that involve autonomic effectors. Stress is a condition in which a homeostatic comparator senses a discrepancy between afferent information to the brain about a monitored variable and a set point or other instructions for responding. The error signal drives effectors including components of the autonomic nervous system in a manner that reduces the discrepancy. Cumulative wear and tear (allostatic load) decreases effector efficiency, eventually precipitating dyshomeostatic vicious cycles. Feed-forward anticipatory processes shift input–output curves determined by the “Regulator.” The **right panel** shows major types of sensory afferent nerves and the corresponding abnormalities in autonomic reflexes observed in heart failure are illustrated. Sympathoexcitatory afferents are shown in **green**; sympathoinhibitory afferents in **blue**. Examples of underlying mechanisms acting at sensory, central, efferent, and effector organ sites that contribute to the reflex cardiovascular/respiratory dysregulation are noted. Aff = afferent; DRG = dorsal root ganglia; HR = heart rate; ParaSNA = parasympathetic nerve activity; SNA = sympathetic nerve activity.

The ANS controls cardiovascular homeostasis. Dysregulation of cardiac autonomic transmission is a hallmark of HF, which is characterized by sympathetic activation and parasympathetic withdrawal (Figure 2). Neural remodeling occurs in parallel with cardiac remodeling and contributes to dysfunction. In canines with pacing-induced HF, increased circulating catecholamines are associated with a loss of noradrenergic nerve terminals in the failing ventricles (26). On the other hand, HF induced by MI may be associated with nerve sprouting and sympathetic hyperinnervation (27,28). The coordination of NE synthesis, release, and removal becomes disrupted, with elevated release and suppressed reuptake leading to high extracellular NE that is detrimental to the heart (29). As the disease progresses, acetylcholine (Ach) replaces NE in some sympathetic neurons, and NPY synthesis and release increase, potentially contributing to pathology (30,31). It is now recognized that neural remodeling occurs throughout the nervous system in HF. In addition to neurochemical plasticity, morphologic changes include remodeling of axon arbors in the heart—both degeneration and regrowth—as well as increased size of cell bodies and dendritic arbors within stellate and intracardiac ganglia (27,28). Electrical remodeling includes enhanced excitability in stellate ganglia (32), altered firing frequencies of sensory afferents (33) and parasympathetic efferents, as well as remodeling of connections within intracardiac ganglia (34). These neural changes influence the heart, causing down-regulation of β1, up-regulation of β2 adrenergic receptors, and modulating cardiac electrophysiology (35). A recent clinical trial showed that propranolol, which blocks both β1 and β2 receptors, is much more effective than metoprolol in managing patients with electrical storm (36). β2 switching takes place, not only on myocytes, but also on human and rat stellate neurons in models of sympathetic hyperactivity (37). The neurotransmitter switching may enhance epinephrine release and lead to greater postsynaptic excitability in disease. Some of the mechanisms controlling neurochemical plasticity and beta receptor signaling are well-described, but little is known about the molecular basis for morphologic or electrical remodeling of the nervous system in HF.

#### Pathophysiology of baroreceptor reflex and cardiac control in HF

Arterial and cardiopulmonary baroreceptor reflexes are major regulators of blood pressure and cardiovascular function (Figure 2). Baroreflex dysfunction contributes to excessive neurohumoral excitation in HF (38,39). Decreased baroreflex sensitivity for control of heart rate predicts cardiac arrhythmias and sudden death post-MI and in chronic HF. Mechanisms operating at multiple sites contribute to baroreflex dysfunction, including decreased compliance of baroreceptor-innervated arteries and heart; impairments in sensory transduction, central nervous system (CNS) mediation of the reflex and efferent neurotransmission; and actions of angiotensin II, aldosterone, and oxidative stress (38, 39, 40, 41, 42). Baroreflex interactions with sympathoexcitatory reflexes are also important (43, 44, 45, 46). Several factors limit our understanding. Cause–effect relationships can be difficult to discern due to bidirectional, positive-feedback (HF → ↓baroreflex sensitivity → ↑HF). The pathophysiology differs in HF of different etiologies, HF with reduced ejection fraction (HFrEF) versus HF with preserved ejection fraction (HFpEF) (47). Few studies have compared baroreflex/autonomic phenotypes in different models of HF. Studies of patients and rodent models of HFpEF implicate nitrosative stress, endothelial dysfunction, and decreased cardiac NAD^+^ in the pathogenesis (48, 49, 50). Baroreflex/autonomic phenotypes were not reported in those studies. The majority of baroreflex studies assess control of heart rate, sympathetic activity, and vascular resistance. Few have examined control of venous compliance and blood volume distribution, important end points in HF (51, 52, 53). Application of new technologies has led to major advances in understanding molecular mechanisms. These technologies have only recently been applied in studies of baroreflex function. Particularly relevant findings are the striking molecular and functional diversity of nodose sensory neurons (7), the identification of mechanosensitive ion channels mediating baroreceptor activation (54, 55, 56, 57), CNS mechanisms of blood pressure sensing that interact with baroreceptor inputs (58,59), and the ability to map and selectively activate or inhibit molecularly targeted neural circuits. These approaches have not yet been used in animal models of HF.

#### Autonomic neural mechanisms in stress-related cardiac pathophysiology

The topic of stress, catecholamines, and acute cardiovascular pathophysiology is difficult but important. The definition of stress is still unsettled. Selye’s theory about stress being the nonspecific response of the body to any imposed demand was refuted more than 20 years ago (60). According to a homeostatic theory (61), stress is a condition where a sensed discrepancy between state information and set points for responding drives effectors to reduce the discrepancy; however, no homeostatic comparators—“homeostats”—have been identified (62). “Distress” can be defined as a form of stress that is conscious, aversive, associated with instinctively communicated signs, involves multiple shifts in input–output relationships in the central autonomic network (allostasis), and augments sympathetic adrenergic activation (Figure 2). Takotsubo cardiopathy, cardiac contraction band necrosis evoked by intracranial bleeding, and MI in disasters (eg, earthquakes) exemplify acute cardiac pathophysiological states where stress and catecholamines likely play important roles. Because of the rapid, unpredicted nature of these events, obtaining scientifically valid data ethically is challenging. Published reports about the magnitude of catecholaminergic activation in takotsubo cardiopathy is inconsistent (63). Concepts about mechanisms of catecholamine-induced cardiotoxicity in these disorders have included a switch in beta-adrenoceptor signaling, dysregulation of intracellular ionized calcium, mitochondrial dysfunctions, toxic catecholamine oxidation products, and catecholaldehydes—typically studied in isolated fashion.

#### Neuromodulation and neurotherapeutics in HF

The ANS becomes imbalanced in HF, with withdrawal of parasympathetic tone and increased activation of the sympathetic nervous system (Figure 2). Pharmacologic therapy with beta-blockers, blockade of renin-angiotensin-aldosterone (RAAS) activation, and more recently, neprilysin inhibition are the mainstays of medical therapy. However, HF prevalence has increased as the population has aged. As a result, concomitant device therapy has received increasing attention in HF with autonomic modulation as 1 important target. There are many device-based approaches in development to modulate autonomic activity, including vagus nerve stimulation, spinal cord stimulation, and baroreceptor activation. Preclinical studies all showed a benefit of each of these approaches, as well as early clinical pilot studies (64, 65, 66, 67, 68, 69, 70, 71). However, larger, randomized studies of spinal cord and vagal nerve stimulation have been disappointing (72,73). The midterm results of baroreceptor activation have been encouraging (71), although the study is continuing to evaluate HF hospitalization and mortality outcomes. Given these clinical results, there is no consensus at present on the role of any of these technologies in the treatment of HF. This likely reflects the complexity of autonomic modulation with many different factors that may affect outcomes, including trial design, stimulation parameter (such as site, frequency, and intensity), and patient population under study. Another important gap in our understanding is the interaction between pharmacologic agents that modulate autonomic activity and the devices noted in the preceding text. In this regard, beta-blockers are ubiquitous in the treatment of HFrEF. Metoprolol, bisoprolol, and carvedilol are the best-studied agents. However, for ventricular arrhythmic control, nonselective beta-blockers without alpha blockage activity such as propranolol or nadolol are more effective, at least for primary arrhythmia disorders (74).

### Knowledge gaps

1. In order to identify the basis for neural remodeling in HF, we need to understand the electrophysiological and morphologic properties of peripheral ganglia in health or disease, and determine the role of immune and glial cells in these processes.
2. For takotsubo cardiopathy, it is necessary to know 1) the long-term consequences of massive, but short-term, catecholaminergic activation; 2) the bases for occurrence mainly in post-menopausal women; 3) the relative roles of adrenaline versus cardiac sympathetic stimulation; and 4) beta-adrenoceptor blockers and recurrence.
3. Our understanding of fundamental mechanisms underlying baroreflex dysfunction in HF (especially HFpEF) is limited, in part due to the paucity of studies using novel and emerging technologies to target specific molecules, neurons, and neural circuits in animal models of HF, most notably HFpEF.
4. As to neuromodulation in HF, understanding the differences and similarities of populations that may respond to vagal nerve stimulation, baroreceptor stimulation, and spinal cord stimulation are not well understood. Also, identifying the optimal timing for initiation of therapy and appropriate trial design for device-based therapies remains unclear.
5. Ascertainment of beta-blocker type best served to target long-term management of arrhythmias in HF is unclear.

### Research priorities

1. Investigate ANS pathophysiology in HF including determining the causes of neural remodeling throughout the cardiopulmonary circuit(s), including the role of neural-immune interactions. Determine relative roles of adrenoceptors and intracellular catecholamines in takotsubo cardiopathy, stroke-related cardiac necrosis, and other forms of acute cardiac pathophysiology.
2. Accelerate application of new and emerging technologies to studies of baroreflex dysregulation in animal models of HFpEF and HFrEF, including studies of sensory, central, and efferent components, and baroreflex interactions with sympathoexcitatory reflexes. Establish optimal stimulation protocols for interventions.
3. Compare different beta-blocker classes in long-term management of ventricular arrhythmias in patients with HF.

## Neuroelectrophysiological Contributions to Atrial Arrhythmias

### Background

**Figure 3:**
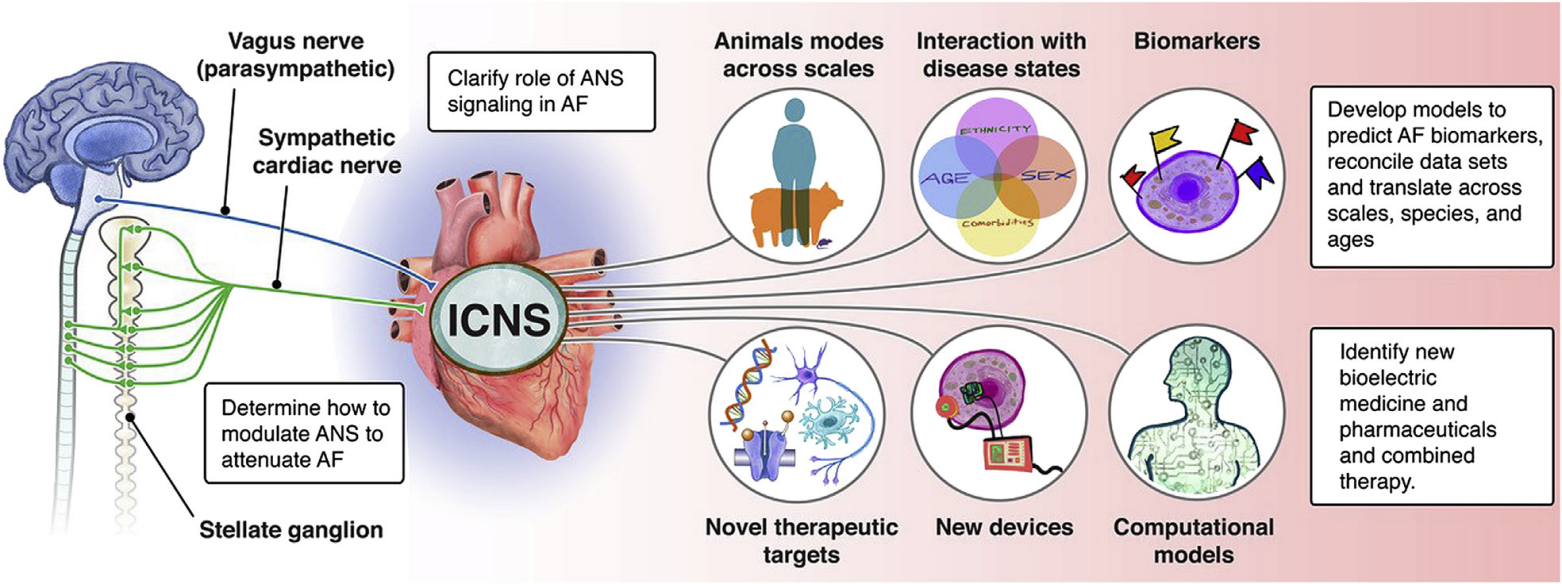
ANS and Atrial Arrhythmias Research opportunities to reveal mechanisms by which the autonomic nervous system contributes to the development and maintenance of atrial arrhythmias. New therapeutic targets of neuromodulation and approaches to neuromodulation are depicted. AF = atrial fibrillation; ANS = autonomic nervous system; ICNS = intrinsic cardiac nervous system.

Decades of research in preclinical animal models, computational models, and human patients have demonstrated that stimulation of the vagus nerves reduces atrial refractoriness, thereby increasing the likelihood of inducing and sustaining an atrial arrhythmia. Atrial fibrillation (AF) is the most common heart rhythm disorder and a major cause of stroke (75,76). Unfortunately, current therapeutic approaches—both pharmacologic agents and AF ablation—are imperfect with risk for procedural complications, untoward side effects, and/or limited efficacy (77). Despite tremendous advances in defining arrhythmogenic substrates and triggers for AF across etiology, our understanding of the precise molecular mechanisms underlying AF is incomplete. Growing evidence supports the critical role for the ANS in modulating onset and progression of AF, especially in the early stages (78,79). However, the precise mechanisms by which the ANS creates a vulnerable substrate for AF are not known. Research opportunities to reveal mechanisms by which the ANS contributes to the development and maintenance of atrial arrhythmias will allow for identification of new therapeutic targets of neuromodulation and new approaches to treatment (Figure 3).

The link between activity of the ANS and atrial arrhythmias has been known for over a century. Recent clinical trials have tested autonomic modulation as a potential therapy for patients with AF (80,81). However, results from these clinical studies have been inconsistent, motivating the need for a better understanding of the mechanistic relationship between state of the ANS and atrial electrophysiology at the cell, tissue, and systems level (78,82,83).

#### Early promise in autonomic control sets stage for new treatment modalities

Preclinical and clinical studies suggest that autonomic modulation can prevent the occurrence of AF. Ganglionated plexi (GP) ablation, so far, is the most studied intervention that reverses acute autonomic remodeling and suppresses AF, but still remains (even after more than a decade of clinical research) a debated Class 2 recommendation in recent guidelines. Moreover, even though GP ablation in addition to pulmonary vein (PV) isolation improved sinus rhythm maintenance at follow-up in some studies, the best technique to identify and ablate GP has not been defined. Moreover, the AFACT (Atrial Fibrillation Ablation and Autonomic Modulation via Thoracoscopic Surgery) study suggested that GP ablation in patients undergoing thoracoscopic surgery for advanced AF did not affect AF recurrence and resulted in more adverse effects (84). A variety of interventions are under investigation including sympathetic modulation via renal denervation, GP ablation, Botox, and CaCl_2_ injection (85). Several surrogate markers of autonomic tone have been revealed in preclinical studies, and these can potentially be used as predictors of the treatment effect of autonomic modulation.

This is important since the current standard technique for AF ablation—PV isolation—is well known to go hand in hand with damage to the intrinsic cardiac nervous system. This is not surprising given that the highest density of nerve endings in the human atria has been described at the PV/atrial junction. Notably, the standard AF ablation procedure, wide-area PV isolation, transects axons transiting from the GP to the PV myocardium, as well as neurons of 2 to 3 major atrial GP (anterior right GP, part of the superior left GP, and part of the inferior right GP [79]). Studies indicating that vagal denervation following AF ablation procedures is associated with lower AF recurrence rates (86) support the notion that injury to some GP tissue and interruption of their axons to PVs may contribute to the success of PV isolation procedures. This accidental modification of the intrinsic cardiac nervous system results in changes to cardiac autonomic control but diminishes mostly over time (up to 1 year following catheter ablation as a consequence of reinnervation and/or nerve sprouting).

Clinically, various pharmacologic and interventional approaches modulating cardiac autonomic control are under investigation targeting both the sympathetic and parasympathetic nervous system. Moxonidine, a centrally acting sympathoinhibitory agent, can lead to a reduction in post-ablation AF recurrence (87). Anatomical GP ablation in addition to PV isolation resulted in better outcomes compared with high-frequency stimulation–guided GP ablation, but this difference may be attributed to significantly more radiofrequency applications delivered in the former group, covering a larger area for each GP. Other interventions like botulinum toxin injection or calcium-induced autonomic denervation additionally paved the way for various clinical studies in the operating theatre.

Low-level vagus nerve stimulation has been shown in preclinical studies to reverse autonomic remodeling and suppress AF. In humans, low-level vagus nerve stimulation prevented postoperative AF in a small randomized clinical trial (88). Moreover, vagus nerve stimulation can be achieved noninvasively via stimulation of the auricular branch of the vagus nerve at the tragus, which results in favorable changes in neural activity in the brainstem and reduces sympathetic activity and increases centrally mediated parasympathetic outflow. Preclinical studies have been recently translated in humans in a series of randomized clinical trials. In a proof-of-concept study in patients with paroxysmal AF, tragus stimulation for 1 hour significantly shortened AF duration and decreased inflammatory cytokines (80). Chronic, intermittent (1 hour daily) tragus stimulation over 6 months significantly decreased AF burden in ambulatory patients with paroxysmal AF compared with sham stimulation (81). However, it should be noted that the response to tragus stimulation was variable across patients, highlighting that although tragus stimulation is an emerging, promising modality for AF, dosing and/or patient selection have yet to be optimized.

Neuromodulation exhibits memory whereby short durations of therapy result in long-lasting effects (81,89,90). The importance of this finding is that it provides the possibility of achieving long-term effects by applying neuromodulation for short periods of time. The minimum duration of neuromodulation that is required to achieve improvement in relevant clinical outcomes remains to be determined. Future research beyond novel catheter-based/minimally invasive approaches should focus on optimization of the stimulation parameters and rigorous patient selection based on appropriate biomarkers in order to maximize the efficacy of neuromodulation.

Importantly, the presence of concomitant diseases such as obstructive sleep apnea (OSA) and central sleep apnea (CSA) may increase the prevalence, incidence, and recurrence of AF (91, 92, 93, 94, 95, 96, 97, 98). Furthermore, treatment of AF, whether by cardioversion or ablation, may be less effective in patients with underlying OSA. Potential mechanisms linking apneic events to genesis of AF include autonomic responses to apnea (both vagal and sympathetic), hypoxemia, hypercapnia, atrial stretch, and systemic inflammation. Whether interruption of 1 or more of these mechanisms, for example, modulation of the autonomic responses by cardiac ganglia ablation (99), renal denervation (100), baroreceptor stimulation (101), and/or transcutaneous electrical stimulation of the auricular branch of the vagus nerve at the tragus (102) in humans, can attenuate the development or recurrence of AF remains to be determined. It is of critical importance to identify appropriate and effective treatments for OSA, because its prevalence continues to increase. Observational studies suggest that effective treatment of OSA has been linked to more prolonged maintenance of sinus rhythm (103). However, definitive evidence of any such salutary effect of OSA treatment on incident or recurrent AF has yet to be demonstrated in randomized controlled outcome studies. In understanding any effect of OSA on AF, it is important to recognize that OSA and AF are both common conditions, and thus may often coexist. AF has multifactorial etiologies. Although OSA may be a comorbidity of AF, this may not necessarily indicate a causal relationship. Hence, if the AF is due to factors other than the comorbid OSA, treating OSA will be unlikely to prevent AF. It is thus important to develop biomarkers identifying AF that is likely secondary to OSA so as to optimize targeted OSA treatment in studies of AF prevention. A similar construct would apply to other common conditions that may cause, and are often comorbid with, AF such as obesity and hypertension.

#### Fundamental mechanistic understanding is needed to drive clinical treatment

At the cell level, it is generally accepted that activation of G-protein–activated Ach-activated K^+^ channels (*I*_K,Ach_) underlies cholinergic regulation of atrial action potentials. By contrast, the role of sympathetic nerve stimulation in regulating atrial excitability and arrhythmias is less clear, although changes in intracellular Ca^2+^ homeostasis and downstream consequences are thought to be critical factors. Critically understudied is the interaction of the parasympathetic and sympathetic nervous system in affecting Ca^2+^ cycling and membrane excitability in atrial myocytes. Because Ca^2+^ cycling is highly sensitive to autonomic manipulation, deeper investigation into excitation–contraction coupling in the atrium is likely to yield important mechanistic insights into AF. In general, the field lacks a clear understanding of how ANS-mediated changes at the cell level manifest at the level of the whole organ due to a wide array of challenges, including heterogeneous distribution of nerve endings and/or cell surface receptors, interplay between vagal and sympathetic inputs, species and sex differences, and complex behavior that spans spatial and temporal scales.

Another key area requiring fundamental investigation is the impact of the ANS on initiation and persistence of atrial ectopy. Although it is known that AF may arise in the pulmonary veins, ectopy can arise in the atria, and it is not known precisely how the ANS contribute to the emergence of AF triggers. Another big unknown in the field is the relative role of the parasympathetic and sympathetic nervous system in the genesis of AF. Even though animal models demonstrate extensive parasympathetic—and to a lesser extent sympathetic—nerve sprouting in the AF atrium (increasing with persistent AF), the molecular mechanisms underlying neural remodeling and how remodeling contributes to formation of electrophysiological substrate for AF are not known. Immunohistochemistry of post-mortem human atrial tissue demonstrated a shift in the pattern of atrial autonomic innervation in persistent AF, with lower density of cholinergic nerves and higher density of adrenergic nerves in patients with persistent AF compared with those with sinus rhythm (104). Lately, gene-based therapies have shown promise in targeting a variety signaling pathways and downstream effectors (ion channels, gap junctions). A similar gene-based approach could potentially be envisioned to selectively inhibit parasympathetic signaling in the atrium, by expressing C-terminal G-protein inhibitory peptides to target Gα_i_ and Gα_i_ signaling (105,106). Last, but not least, it is unclear how neuromodulation can be effectively accomplished in the atrium with the goal of treating AF. Emerging studies may begin now to provide critical insights (16).

A new and exciting avenue of investigation centers on the role of glial cells as potential biomarkers and possible novel therapeutic targets in AF. In several species, including human, glial cells have been found to be involved in several essential neuronal functions from development to modulating neuronal activity and regeneration after damage. Early evidence supports that this holds true for glia cells within the heart. Moreover, in vitro and in silico models support that they can even directly modulate excitation–contraction coupling in the heart. Although peripheral glia originate from Schwann cell precursor and differentiate in subtypes associated with a variety of neuronal types and functions (including myelinating and nonmyelinating glia), neither have been sufficiently studied in the heart. Recently, it has been demonstrated that S100B-expressing glial cells are widely distributed throughout the heart and its nerves (107). The release of S100B from cardiac glia upon catheter-based treatment of AF has been described to be a hallmark of acute neural damage that contributes to nerve sprouting and can be used to assess damage of the intrinsic cardiac nervous system. Moreover, intracardiac transplantation of Schwann cells—which are known to exert repair functions by providing physical supports to growing axons, ensheathing and myelinating these axons, and producing a diversity of cell adhesion molecules, extracellular matrix molecules, and neurotrophic factors—have been described as a cell-based therapy that can improve HRV and prevent ventricular arrhythmias in rats following MI (108). Taken together, this early evidence suggests that a deepened understanding of the glial anatomy and physiology within the heart appears to be a promising interface to protect atrial electrophysiology in health and disease.

### Knowledge gaps

1. Need for accurate biomarkers as indicators for neuromodulation therapy and response to therapeutic ablation/modulation of ANS (including the GP) in AF.
2. The intersection of concomitant diseases and comorbidities including CSA or OSA needs further study.
3. Lack of adequate studies on the intersection of race, ethnicity, sex, and gender in both risk stratification and treatment of AF.
4. Need for information on how regional variation in innervation and cell surface receptor expression impacts atrial arrhythmogenesis and how this inherent heterogeneity changes with disease.
5. Need for methods to support translation of experimental/computational data from single-cell studies across spatial and temporal scales to draw conclusions about arrhythmogenesis.

### Research priorities

1. Studies to clarify the role of parasympathetic and sympathetic signaling and coexistent disease states in the emergence and maintenance of AF and to identify novel therapeutic targets.
2. Determine how to perform targeted modulation of the parasympathetic and/or sympathetic nervous system in the atrium and design devices, with the goal of attenuating electrical remodeling in AF.
3. Development of modeling and simulation approaches for predicting underlying AF biomarkers, for prediction, reconciliation of data sets and translation of data across scales, species, and ages, and to identify candidates for neurointervention via bioelectric medicine and pharmaceuticals and combined therapy.

## Neuroelectrophysiological Aspects of Ventricular Arrhythmias

### Background

#### Autonomic control of ventricular electrophysiology

**Figure 4:**
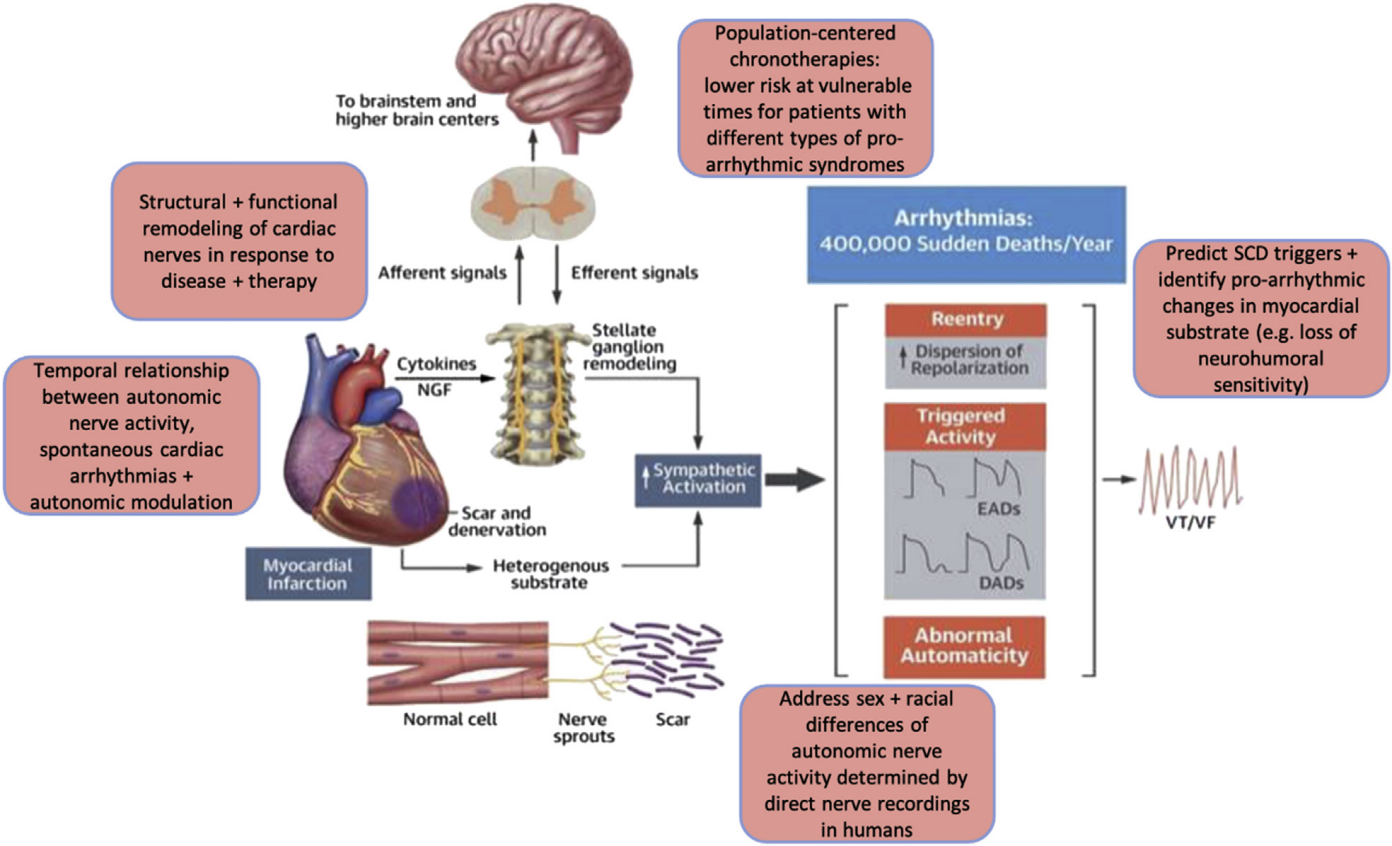
Neurophysiological Aspects of Ventricular Arrhythmias Overview of the neuromyocardial interplay and its impact on ventricular electrophysiology and arrhythmogenesis. Key features of sympathetic ventricular control are highlighted. Remodeling of the parasympathetic nervous system in cardiovascular disease has received significantly less attention (compared to sympathetic remodeling) and may also represent a therapeutic target. Research priorities with the need to address: 1) structural and functional neuronal remodeling; 2) temporal relationship between nerve activity, arrhythmia and autonomic modulation; 3) sex and racial differences; 4) population/patient-centered chronotherapies/lifestyle modification; and 5) reliable prognostic indicators are summarized. Modified from Goldberger et al (202). DAD = delayed after depolarization; EAD = early after depolarization; NGF = nerve growth factor; SCD = sudden cardiac death; VF = ventricular fibrillation; VT = ventricular tachycardia.

Sudden cardiac death (SCD) describes the abrupt onset of ventricular arrhythmias that kill more Americans than any other disease. These life-threatening changes in electrical activity are frequently associated with underlying pathological conditions such as HF. Cardiac neural control occurs primarily via parasympathetic output from the vagus nerve and sympathetic output via the intrathoracic extracardiac ganglia, which pass through the hilum of the heart (where most cardiac nerves are intertwined) along the great vessels (109,110). Three major sympathetic nerves dominate innervation of the ventricles (right and left coronary cardiac nerves and the left lateral cardiac nerve). Both atrial and ventricular GP have been found to modulate ventricular electrophysiology and arrhythmogenesis. Sympathetic activity increases cardiomyocyte cAMP, leading to phosphorylation of several ion channels and Ca^2+^ handling proteins, which typically shortens action potential duration (depending on species) and thereby refractory periods. Early afterdepolarizations may also be induced, leading to increased dispersion of refractoriness and heterogeneity of repolarization (Figure 4). Parasympathetic activity inhibits cAMP generation and therefore tends to have opposite effects on ventricular electrophysiology (prolonged action potentials, decreased amplitude of Ca^2+^ transients, and diminished risk for ventricular arrhythmias) (in most patient populations) (111).

The effects of classical neurotransmitters (ie, NE, Ach) on ventricular electrophysiology have been well studied. Yet, in the setting of cardiovascular diseases (CVD) such as MI or HF, remodeling of the ANS results in changes to cardiac nerve density, distribution, activity, and neurotransmitter/neuropeptide phenotype. With regard to sympathetic remodeling, both hypo- and hyperinnervation may occur, leading to heterogeneous electrophysiological responses and chronic changes to cardiomyocyte adrenergic responsiveness. Together, these responses can provide the trigger and substrate for potentially lethal ventricular arrhythmias (112). Recent studies show increased release of NPY in MI and HF, and elevated NPY is a predictor of post-MI ventricular arrhythmias (113). Further, NPY independently leads to proarrhythmic changes to ventricular action potentials and calcium transients in isolated rat hearts (113). MI and HF also cause cholinergic transdifferentiation, whereby the sympathetic nerves make and release Ach in addition to NE. Although very little is known about the functional impact of transdifferentiation, recent evidence shows that corelease of Ach may counteract some of the detrimental effects of heterogenous NE release following MI to reduce action potential duration heterogeneity without affecting contractility (114). The electrophysiological consequences of remodeling of the parasympathetic nervous system in CVD have received less attention (compared with sympathetic remodeling) and may represent an important area for therapeutic opportunity.

#### Assessment of ANS function in ventricular arrhythmia

Tonic and phasic autonomic nerve activities are important in cardiac arrhythmogenesis. Consequently, the assessment of ANS function is of growing interest. HRV analyses are the most commonly used noninvasive methods to measure autonomic tone, but there are many limitations including the high prevalence of sinus node dysfunction in HF and AF. The same holds true for autonomic reflex testing following endogenous autonomic stressors (postural challenge, isometric handgrip, cold face/hand immersion, medical oxygen inhalation, and others). Imaging modalities such as iodine-123 metaiodobenzylguanidine (^123^I-MIBG) assessing sympathetic nerve distribution by quantifying local noradrenaline reuptake have also been used to understand the importance of altered sympathetic innervation in cardiac arrhythmogenesis. A myriad of targeted radiotracers have been used for imaging various components of the sympathetic and parasympathetic signal cascades, but their use is limited to relatively few specialized centers. Microneurography is the standard method for sympathetic nerve activity (SNA) recording. Direct recordings from peripheral autonomic ganglia including the intrinsic cardiac nervous system have been used in several animal models (115). However, these methods are invasive and also require significant technical expertise. Recently a new method (neuECG) has been developed to simultaneously and noninvasively measure electrocardiogram (ECG) and skin SNA in humans over a prolonged period of time (116,117). Neither microneurography nor neuECG can be used to measure parasympathetic nerve activity. Beyond the potential advantages of each of these methods, patient-specific characteristics, including sex and race differences, as well as the dynamic nature of autonomic control during the development and progression of CVD, need to be considered. Genome-wide association studies might be useful to identify genetic variants associated with variables describing autonomic tone with respect to ventricular arrhythmias and their relationship with other arrhythmias.

There are significant sex and racial/ethnic differences of cardiac arrhythmia prevalence. Significant sex differences of cardiac ion channel function may in part underlie the differential prevalence of atrial and ventricular arrhythmias in men and women (118). AF is more frequent in White than in Black Americans (119). HRV analyses show that Black and women examinees have a lower low-frequency, higher high-frequency, and higher high/low-frequency ratio than Whites (120), suggesting that reduced sympathovagal balance may play a role in sex and racial differences of AF prevalence. J-wave syndrome primarily affects men and rarely women (121). Because cardiac ion channel function is controlled by the autonomic tone, it is likely that the autonomic nerve activity plays an important role in the sex and racial difference of QT interval, J-wave syndrome, AF, and other diseases.

#### Circadian variations of ventricular tachyarrhythmias and SCD

The term *circadian* is commonly used to describe processes occurring in cycles of ∼24 hours. Clinical studies that report “circadian” variation of tachyarrhythmias and SCD use this term to describe changes in the frequency of events based on local clock time. Thus, the 24-hour pattern in the frequency of events is influenced by internal or endogenous circadian rhythms, time-of-day–dependent changes in behavior, and time-of-day changes in the environment. A 24-hour pattern in the incidence of SCD was initially reported in the Framingham Heart Study (122) and later reproduced in large prospective community studies (123,124). These studies show that SCD has a nighttime nadir followed by a morning and smaller evening peak. Subsequent studies show that the 24-hour pattern in SCD or life-threatening events is different in people living with certain types of CVD. People with HF tend to have an early morning dip followed by an increase in the incidence of SCD and tachyarrhythmias during the daytime hours (125). People with hypertrophic cardiomyopathy have a bimodal distribution in the incidence of SCD that peaks in the morning and again in the evening hours (126).

Different 24-hour patterns in the incidence of arrhythmia-related events also occur in people living with different types of genetic arrhythmia syndromes. For example, studies suggest people living with Brugada Syndrome tend to experience most of their ventricular events between 12:00 am and 6:00 am (127). This pattern is thought to reflect increased vagal activity and withdrawal of sympathetic activity at night. By contrast, pediatric patients living with catecholaminergic polymorphic ventricular tachycardia have ventricular events that are more likely to occur in the afternoon and evening hours (128). The lack of a morning peak in this cohort suggests factors in addition to adrenergic stimulation impact the incidence of arrhythmias in this cohort. There are also different 24-hour patterns in ventricular events in people living with the same arrhythmia syndrome. Three genes are definitively associated with typical long QT (LQT) syndrome: *KCNQ1* (LQT1), *KCNH2* (LQT2), and *SCN5A* (LQT3) (129). People with LQT1 tend to experience cardiac events between 12:00 pm and 6:00 pm, people with LQT2 typically experience cardiac events between 6:00 am and 12:00 pm, and people with LQT3 do not show a clear time-of-day difference in the incidence of events (130). The time-of-day difference in the cardiac events in people with LQT1 and LQT2 likely reflects gene-specific differences in triggers for arrhythmias. People with LQT1 tend to experience cardiac events with exercise, whereas people with LQT2 typically experience events with emotions (eg, fear, anger, sudden noise, or startle) (131). The existence of distinct 24-hour patterns in tachyarrhythmias and SCD in populations living with different types of CVD suggest there are disease-specific arrhythmogenic risks associated with certain day/night rhythms in behaviors, the environment, and/or endogenous circadian rhythms (132). As clinician scientists separate which of these factors contribute to the different 24-patterns in SCD, they can target each of these factors to develop tailored therapeutic strategies that mitigate the risk of SCD across the 24-hour cycle in different subpopulations (133).

#### Autonomic neuromodulation and neurotherapeutics for ventricular arrhythmia and prevention of SCD

The life-threatening changes in electrical activity preceding SCD are often linked to acute and chronic changes in sympathetic and/or a parasympathetic structure and function, which has been most extensively studied in myocardial ischemia and related ischemic cardiomyopathy. This includes denervation and reinnervation/nerve sprouting in infarcted areas, as well as those being remote from myocardial scarring (134). Related ventricular arrhythmias include premature ventricular contractions, (monomorphic, pleomorphic, and polymorphic) ventricular tachycardia, and ventricular fibrillation, which are mechanistically linked with reentry, triggered activity, and abnormal automaticity.

Although changes in the ANS of the heart pose their own inherent risks, responses occurring at the level of the individual ventricular myocyte can also contribute significantly in the presence, but also absence of obvious structural heart disease. This point is illustrated by genetic mutations associated with catecholaminergic polymorphic ventricular tachycardia and certain forms of LQT syndrome that lead to arrhythmias ultimately triggered by changes in autonomic tone. There is evidence that other less well-characterized changes in ventricular myocyte responses may also contribute to arrhythmogenesis. These include: 1) disease-induced changes in cellular organization that alter compartmentation of the cAMP/PKA signaling pathway, which is essential for orchestrating regulation of multiple mechanisms affecting electrical activity; 2) effects of sympathetic and parasympathetic cotransmitters, such as NPY and vasoactive intestinal peptide, the increased release of which corresponds with an increased incidence of SCD; and 3) cellular responses to dynamic changes in sympathetic and/or parasympathetic tone, which may contribute to the increased incidence of SCD in the absence of structural changes, such as those occurring in OSA and epilepsy. Conversely, increased parasympathetic tone is associated with a decreased incidence of ventricular arrhythmias and SCD in most populations, yet the cellular and molecular basis for this effect and its importance during long-term follow-up are still poorly understood. A better appreciation of the mechanisms that contribute to both pro- and antiarrhythmic effects of sympathetic and parasympathetic stimulation on ventricular myocytes is likely to facilitate the development of novel therapeutic strategies for preventing life-threatening changes in electrical activity associated with SCD (135).

HF, a common clinical condition associated with ventricular arrhythmias, is characterized by down-regulation of beta-1 and up-regulation of beta-2 receptors (35). Chatzidou et al (36) showed that in patients with electrical storm, propranolol is much more effective than metoprolol in suppressing ventricular arrhythmias. Whether or not propranolol is better than metoprolol in long-term management of HF remains unknown. Moreover, the optimal sympathicolytic therapy for the treatment of electrical storm and chronic treatment of ventricular arrhythmias has to be defined. Preclinical and clinical approaches under investigation to directly or indirectly interfere especially with left ventricular neural control include sympathetic/renal denervation, spinal cord stimulation, and intracardiac nerve stimulation (136).

Whether stellate ganglion block or left/right cardiac sympathetic denervation is superior to propranolol in managing patients with electrical storm is not known. In the future, direct measurements of autonomic nerve activity before and during ventricular arrhythmias by using miniaturized devices that combine sensing, stimulation, and recording modalities for scientific and therapeutic closed-loop applications might spur research priorities. Therefore, the effects of neuromodulation methods (including various protocols of intermittent and continuous neuromodulation) on nerve activities (electrophysiological and molecular mechanisms) and neuromorphology (neurotrophic effects, nerve [re-]growth) needs to be explored. The selection of a targeted intervention and dosing depending on patient history (including individual autonomic cardiac control) should be considered and may be more effective, than a “one-size-fits-all” neurotherapeutic intervention (137).

### Knowledge gaps

1. Structural and functional remodeling of cardiac sympathetic and parasympathetic nerves in response to CVD and neuromodulating therapies are under investigation. These include the effects of cotransmitters and neuropeptides on ventricular cardiomyocyte signaling and resulting ventricular electrophysiology.
2. The temporal relationship between autonomic nerve activity, spontaneous cardiac arrhythmias and their responses to beta-blockers and other autonomic modulating drug, interventional, and device-based therapies is not known.
3. The sex and racial differences of autonomic nerve activity determined by direct nerve recordings in humans.
4. Population/patient-centered chronotherapies/lifestyle modification to lower the risk of events at vulnerable times for patients with different types of proarrhythmic syndromes (acquired, congenital).
5. Reliable prognostic indicators that predict increased frequency of SCD triggers and/or identify proarrhythmic changes in the myocardial substrate (eg, loss of neurohumoral sensitivity).

### Research priorities

1. Assessment of cardiac sympathetic and parasympathetic nerve structure and function in ischemic and nonischemic CVD, including nerve density, distribution, functional neurotransmitter/neuropeptides throughout the time course of disease, and the interaction with other cell types, for example, cardiac glial, immune cells, and adipocytes.
2. In vitro assessment of how changes to nerve structure and function (including cotransmitters) impact ventricular cardiomyocyte signaling, ion channel function, action potentials, and calcium transients in both acute and chronic settings while integrating experimental and computational studies to bridge gaps between cellular and organ-level electrophysiological function and arrhythmia risk.
3. Investigate individual and population specific autonomic nerve activity (by considering sex and racial differences as well as relationship with circadian rhythms) and their response to neurotherapeutic interventions in randomized controlled trials.

## Autonomic Nervous System Alterations in Cardiopulmonary-Related Sleep Disorders

### Background

#### Diurnal and circadian influences on cardiovascular function

Multiple aspects of the cardiovascular system display prominent diurnal variation due to the influences of circadian rhythms, sleep–wake states, and sleep disorders (particularly sleep apnea but also periodic limb movements) and associated changes in ANS function. Specifically, diurnal variations in cardiac electrophysiology (including PR and QT interval length, heart rate, HRV, propensity for atrial and ventricular arrhythmias, and ion channel activity), blood pressure, cardiac output, MI, stroke, and SCD are well described and are at least partially mediated by the ANS (138, 139, 140, 141, 142, 143). Heart rate and rhythm, blood pressure, and cardiac output are regulated by the sympathetic and the parasympathetic nervous systems through circulating neurohumoral factors (cortisol, catecholamines) and proinflammatory cytokines that display circadian rhythmicity. Atrial arrhythmias are most likely to occur during the night when vagal tone is highest, whereas ventricular arrhythmias are more likely to occur during the early morning when sympathetic activity increases. Blood pressure typically declines by at least 10% at night relative to the day, attributable to increased parasympathetic activity during sleep. OSA may obliterate (or even reverse) this “dipping” pattern due to the sympathetic surges that occur with obstructive respiratory events (144). Although the mediating mechanisms linking circadian and sleep–wake states and sleep disturbances to cardiac physiology involve autonomic pathways, the specific mechanisms underlying these associations are only partially understood. An improved understanding of the roles of circadian and sleep physiology in cardiopulmonary disease may enhance the identification of at-risk populations, vulnerable time periods for adverse cardiac events, and optimal times for administration of chronotherapy-directed interventions. Specific elucidation of the roles of ANS alterations that occur with disorders of circadian rhythms, sleep–wake states, and sleep disorders may further help identify novel therapeutic targets and interventions.

#### Circadian physiology: influences on the heart and roles of the ANS

The circadian system, comprising a master central clock within the suprachiasmatic nucleus of the hypothalamus and of peripheral clocks located within multiple tissues, governs an array of physiological oscillations that occur over 24-hour periods. Transcriptional/translational negative feedback loops of the core clock machinery (*CLOCK, BMAL1, PERIOD, CRYPTOCHROME*) result in rhythmic oscillations in gene expression, ion channel function, and neurohumoral signaling (including autonomic function), influencing multiple physiological systems (145). Circadian rhythms generate and/or influence a broad spectrum of ANS patterns that can affect blood pressure, coronary artery perfusion, atherosclerosis, cardiac function, and CVD risk factors (obesity, diabetes) (142,146,147).

Most studies of the role of the circadian rhythms and the ANS have focused on the effects of circadian variations in ANS activity on ECG-based parameters (143,148, 149, 150). Both the central circadian pacemaker and a peripheral clock within the heart influence cardiac circadian rhythms that modulate the biophysical properties of major cardiac ionic channels, ionic conductance, and calcium overload (138,151). The central circadian clock directly influences propensity for arrhythmias via the ANS and other neurohumoral signaling, whereas a local circadian clock in the heart—under control of the central pacemaker—may drive a circadian rhythm in the expression of ion channels in the heart, influencing the arrhythmic substrate (138,151, 152, 153). The relative contributions of the central versus the peripheral clocks and the extent to which their effects on the cardiovascular system are mediated by the ANS, however, are not clear. Based on results from experiments using autonomic blockade and cardiac denervation studies, Black et al (138) suggest that the ANS influences the circadian rhythm in the heart by synchronizing the local cardiac clock to drive circadian oscillatory gene expression. Modulation of the cardiomyocyte molecular clock may provide novel therapeutics but has not been well investigated.

The relevance of circadian rhythms in the pathogenesis of CVD and as an avenue for developing new treatment strategies is supported by the range and prevalence of factors that adversely affect circadian rhythms in the population. Specifically, exposures and disorders that impact circadian alignment may also increase CVD. Environmental stimuli (light, temperature), internal cues (eating, exercise, sleep), social conditions (shift work), and health conditions (obesity, diabetes) modulate circadian output through neurohumoral signaling. Inopportune light, unhealthy behaviors, work-related stressors, and metabolic disorders, therefore, may result in circadian misalignment and contribute to CVD. Variants in clock genes associate with a broad range of cardiometabolic, as well as sleep, disorders, which can further directly exacerbate CVD or via ANS effects (147,154, 155, 156). In addition, given that circadian signaling is at least partially mediated by the ANS, disorders that affect the ANS may impact cardiovascular health via effects on circadian rhythms.

Challenges for analysis of circadian patterns of autonomic neural biomarkers include the duration of observation—from 1 to several days; the need to define standard methods of data acquisition, processing, and timing of measurements; and the link between the studied parameter and outcomes of interest. Assessing for important, but subtle, deviations from normal circadian variation can be challenging depending on the approach used and is influenced by whether the environment is natural or controlled. In addition, linking abnormalities in circadian variation to an outcome such as SCD may necessitate evaluation of the effects on ventricular electrophysiology (repolarization [149], refractoriness [143]) rather than on the sinus node, unless they are highly correlated. Similarly, because of different innervation patterns, circadian effects on the atrium and vascular system may not be correlated with those of the sinus node. Furthermore, there may be other variations with longer periodicity (day of the week, season) that need to be accounted for.

#### Sleep–wake states: background for ANS–cardiovascular interactions

Understanding the influences of normal sleep–wake physiology and disturbed sleep is important for appreciating the role of the ANS on cardiopulmonary function. Integration of cardiorespiratory control during sleep is achieved at multiple levels within the neuraxis (157). Pontine and suprapontine, as well as cerebellar mechanisms, can influence cardiorespiratory regulation during sleep and waking. Sites within the cerebral cortex also play major roles, particularly in modulating sympathetic and parasympathetic outflow and breathing patterns.

Sleep consists of repetitive cycles of non-rapid eye movement (NREM) and rapid eye movement (REM) states over the sleep period. Each sleep state is characterized by distinct levels of ANS activity and physiological changes in cardiovascular and respiratory functions. At the beginning of sleep, NREM sleep predominates and is accompanied by a progressive decline in SNA and increase in parasympathetic activity that result in a reduction in blood pressure and heart rate. The impact of SNA on cardiovascular function is decreased by more than one-half from wakefulness to stage N3 of NREM sleep. The autonomic stability of NREM sleep, with the attendant mild hypotension, bradycardia, and reduced cardiac output and systemic vascular resistance, provides a relatively restorative neurohumoral background. The occurrence of bradycardia appears to result mainly from enhanced vagus nerve activity, whereas the hypotensive response appears to be primarily due to decreased effects on sympathetic vasomotor tone. During transitions from NREM to REM sleep, bursts in vagus nerve activity may produce pauses in heart rhythm and occasional brief periods of asystole. REM sleep occurs at ∼90-min intervals, subserving the brain’s neurochemical functions and behavioral adaptations. This dynamic state, however, can disrupt cardiorespiratory homeostasis. In general, the brain’s increased excitability during REM sleep can result in major surges in SNA to the heart and coronary vessels. In individuals with ischemic heart disease, the strong bursts in sympathetic activity can cause arrhythmias and even SCD (158). During REM sleep, baroreceptor gain is reduced and is a factor that contributes to the reciprocal pattern of increased sympathetic activity and reduced parasympathetic tone. Consequently, heart rate can fluctuate markedly, with episodes of tachycardia and bradycardia. REM sleep is also associated with irregular breathing patterns that can lower oxygen saturation, especially in individuals with pulmonary or cardiac disease (159).

In summary, there are well-known changes in normal autonomic function during sleep. Sleep is generally considered to be a protected period, when the cardiovascular system benefits from the restorative influences of the sleeping brain. However, the dynamics of cardiovascular control during sleep can tax the capacity of the diseased coronary circulation and myocardium with surges in sleep-state–related autonomic activity and disruptions in airway function and CNS regulation. In this regard, sleep may constitute an autonomic stress test for the heart (160). Sleep disorders also disturb normal ANS function during sleep, causing cardiovascular stresses. This functional stress test has relevance in a wide variety of conditions, including in HF, with risk for decompensation and atrial and ventricular arrhythmias; in channelopathies, including the long QT3 and Brugada syndromes; and in epilepsy, with enhanced risk for sudden unexpected death in patients with epilepsy.

#### Sleep disorders: perturbations in ANS function impacting CVD; refining sleep disordered breathing phenotypes

Sleep apnea, characterized by recurrent episodes of breathing pauses, sleep fragmentation, and intermittent hypoxemia, increased risk of MI, HF, stroke, arrhythmias, and mortality with effects that are at least partially mediated by the ANS (161). Sleep apnea–associated intermittent hypoxemia and hypercapnia activate peripheral and central chemoreceptors, respectively, leading to increased sympathetic nervous system activity (SNSA) and large ventilatory oscillations, exacerbating cardiac dysfunction and promoting arrhythmogenesis (162,163). A bidirectional association between heart disease and sleep apnea is also likely: cardiac dysfunction leading to prolonged circulatory time can promote ventilatory oscillations, leading to apneas, hypoxemia, and SNSA. The interactions of hypoxia with the cardiorespiratory system and ANS, however, are complex, reflecting multiple molecular sensing and transduction pathways, including arterial chemoreceptor stimulation and secondary neurotransmitter release, reactive oxygen species generation in the mitochondria, hypoxic inducible factor-1–stimulated gene expression, respiratory-related autonomic modulation of electrophysiological action potential duration (163) and cross-talk among these systems (164,165). Moreover, these processes are sensitive to the severity and patterns of hypoxia, which vary substantially among patients with sleep apnea. Autonomic dysregulation (AD) during OSA involves complex changes in sensory feedback from somatic and visceral sites. During AD, central autonomic control system excitability becomes altered causing sympathoexcitation, hypertension, and arrhythmias. Central autonomic network excitability is influenced by many afferent systems, including baroreceptors, central and carotid chemoreceptors, and airway and other visceral afferents. The detailed makeup of this control system during OSA is not fully understood (166). For example, carotid sinus denervation is only partially effective in resolving AD in OSA, and gut dysbiosis has been implicated in OSA-induced hypertension in recent work (167). Little attention has been paid to upper airway afferent feedback in OSA-induced hypertension. Multielectrode array recordings from neurons in the central autonomic control network suggest that it is sparsely excited during increased baroreceptor input and that synaptic inhibition dominates circuit function. OSA-induced sensory feedback may induce sympathoexcitation in this network by reducing inhibition (disinhibition).

Additional several potential targets for reducing SNSA and improving breathing, some of which interact, including improving cardiac function and optimizing heart rates. Neuromodulatory strategies that enhance inhibition within the central autonomic control network have potential for improving AD in OSA. A promising neuromodulation therapy—hypoglossal nerve stimulation—significantly improves OSA in selective patients (168). The extent to which this therapy actuates upper airway afferent systems that directly modulate excitability of the central autonomic network is unknown. Supplemental oxygen can decrease carotid body chemoreflexes, mitigate loop gain and improve oxygenation, but has not been studied rigorously for treatment of sleep disordered breathing (SDB) (but is the focus of a randomized controlled trial of CSA and HF: The Impact of Low Flow Nocturnal Oxygen Therapy on Hospital Admissions and Mortality in Patients With Heart Failure and Central Sleep Apnea [LOFT-HF]; NCT03745898). The role of positive airway pressure needs to be evaluated in patients well characterized according to ANS function and physiological endotypes (the failure of prior positive airway pressure trials may reflect the failure to enroll groups with treatment-responsive phenotypes). Novel upstream targets for treatment of SDB may include the carotid body and reactive species generation at the carotid body/brainstem levels; downstream targets include beta blockage. SDB phenotypes vary by sex (169), and gender/sex-related factors may influence susceptibility of cardiac disease; therefore, studies need to carefully consider sex/gender biology. There is a need to consider the associations of SDB with both HFrEF and HFpEF, given animal data indicating modifications of ventilatory-coupled SNSA across these settings.

Current clinical practice focuses on quantifying SDB severity, characterizing both OSA and CSA burden, based on a count of breathing pauses (Apnea Hypopnea Index), which does not characterize the variations in SDB physiology or between-patient heterogeneity, which likely include wide variation in autonomic regulation (170). Understanding of the role of the ANS in both the etiology of SDB and its impact on heart disease requires enhanced physiological phenotyping of SDB—that is, subtyping patients according to their autonomic response to apneas as well as their predominant mechanisms for SDB: loop gain, arousal threshold, circulatory delay, and neuromuscular collapsibility. For example, a recent study showed that the heart rate response to apneas and hypopneas predicts cardiovascular morbidity and mortality more so than the Apnea Hypopnea Index and other traditional SDB metrics (171).

Other sleep disturbances also can trigger the ANS and contribute to CVD. Periodic limb movements can trigger large sympathetic and blood pressure surges, and in the long term, are associated with increased risk for daytime hypertension and CVD (172,173). Insomnia and other disorders characterized by sleep fragmentation also lead to SNSA and are associated with CVD (174). However, the roles of treating these disorders as a strategy for CVD risk reduction or for treating associated AD have not been systematically assessed.

**Figure 5:**
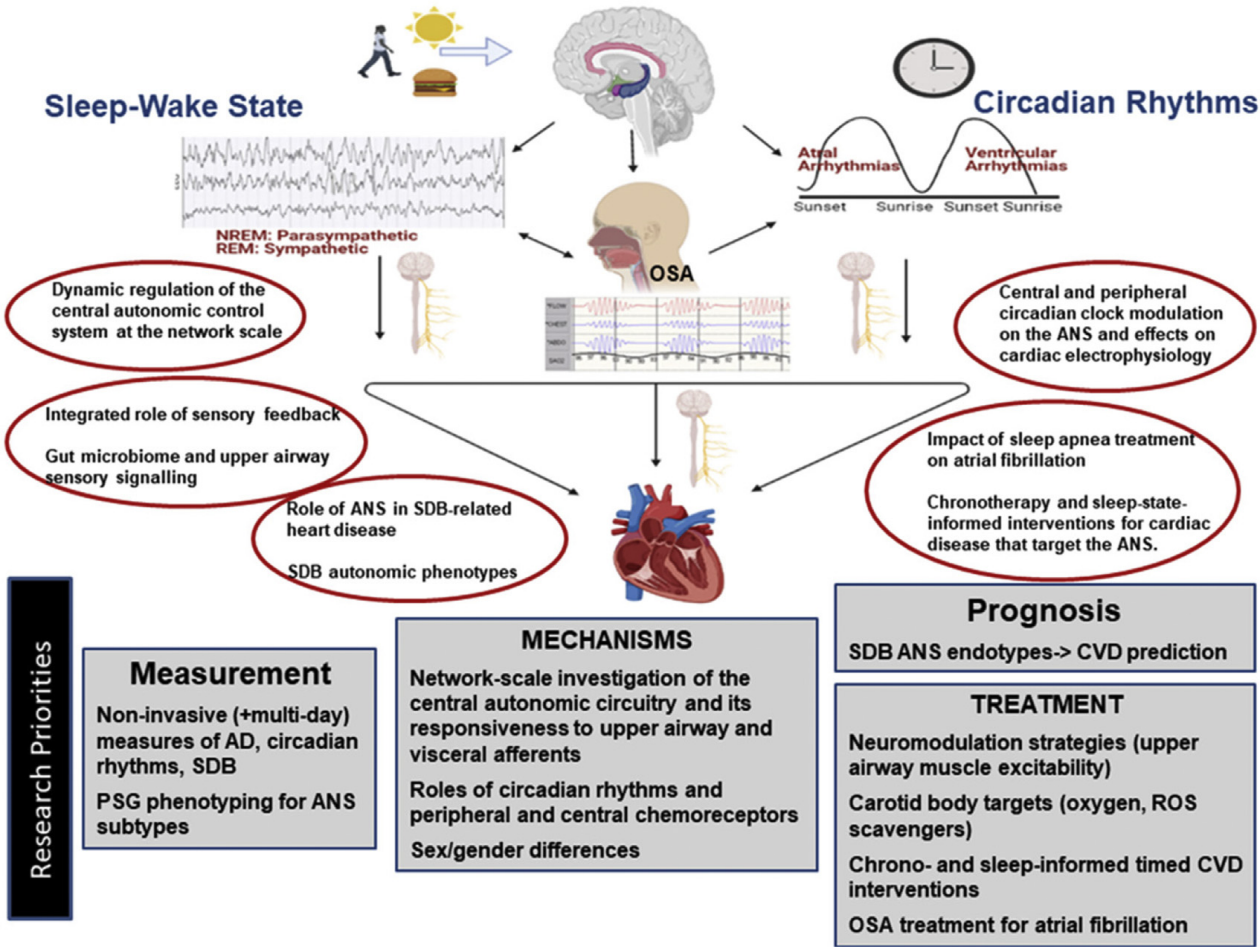
ANS Alterations in Cardiopulmonary-Related Sleep Disorders Autonomic nervous system (ANS) function is influenced by sleep–wake **(left)** and circadian **(right)** rhythms. Obstructive sleep apnea (OSA) is influenced by the ANS (although to a variable degree according to specific endotype) and also alters ANS function. Autonomic dysfunction (AD) resulting from these factors influence cardiovascular function, including the time predilection for arrhythmias, diurnal blood pressure patterns, and cardiac events. Knowledge gaps (in **circles**) reflect the need for an improved understanding of the interactions of sleep, circadian and cardiovascular processes, and mediating roles of the ANS. Diagnostic, prognostic, mechanistic, and treatment needs follow these gaps. CVD = cardiovascular disease; NREM = non-rapid eye movement; PSG = polysomnography; REM = rapid eye movement; ROS = reactive oxygen species; SCD = sudden cardiac death; SDB = sleep disordered breathing.

A summary of the ANS alterations association with sleep and circadian processes and the related knowledge gaps and research priorities are depicted in Figure 5.

### Knowledge gaps

1. Knowledge of how central and peripheral circadian clocks modulate the ANS and effects on cardiac electrophysiology, as well as understanding the dynamic regulation of the central autonomic control system at the network scale, including the integrated role of sensory feedback from the carotid body, baroreceptors, airways, and other sites in controlling the excitability of the autonomic control system, and their influence on dynamic networks that shape interactions among sleep, respiration, and cardiac function is needed.
2. The role of chronotherapy-informed and sleep-state–informed interventions for cardiac disease that target the ANS is unclear.
3. Understanding the role of the ANS in both the etiology of SDB and its impact on heart disease, and using this knowledge to better characterize SDB phenotypes is needed, that is: a) specifying which phenotypes reflect underlying AD and predisposition to cardiac disease; b) specifying phenotypes reflecting predisposition to AD that mediation of cardiac disease; and c) harnessing enhanced phenoptyping methods that scale and are validated; for example, using signals from devices, wearables, and polysomnography (ie, subtyping patients according to their autonomic response to apneas as well as their predominant mechanisms for SDB: loop gain, arousal threshold, circulatory delay, and neuromuscular collapsibility).
4. Identifying the role of intermittent hypoxemia and/or hypercapnia on ANS function and heart disease and understanding the mechanisms by which intermittent hypoxemia and hypercapnia lead to augmented SNSA and potential modulation by the central and peripheral chemoreceptors. Elucidating the mechanisms by which SNSA is coupled with respiratory signaling, and influence heart disease. Understanding of the impact of sleep apnea on AF susceptibility and the implications for apnea management in the context of AF ablation.
5. The role of gut microbiome and upper airway sensory signaling in regulating long-term autonomic excitability with specific reference to OSA is unclear.

### Research priorities

1. Improved measurement/assessments
  a. Improve the specificity of noninvasive measures of SNA (ie, HRV measures do not adequately represent the sympathetic component) and identify better ECG indicators of atrial and ventricular arrhythmia vulnerability, rather than relying on spontaneous arrhythmia.
  b. Improve noninvasive (and multiday) measurement of AD, circadian rhythms, and SDB physiological phenotypes from multiday wearables and other noninvasive sensors. Leverage information from heart rate, rhythm, oxygen saturation, and activity monitors (used clinically or integrated into wearable technology) for multiday use to noninvasively measure ANS function, sleep disorders, and circadian rhythms in real-world settings to elucidate sleep and circadian influences on CVD, and identify the contextual factors that mediate or moderate these influences.
  c. Better utilize information from clinical polysomnography to develop/test advanced signal processing approaches to identify subgroups with SDB and other sleep/circadian disorders who are at risk for abnormal ANS control and CVD.
2. Mechanisms
  a. Conduct network-scale investigation of the central autonomic circuitry and its responsiveness to upper airway and visceral afferents in preclinical models of OSA.
  b. Identify intermediate mechanisms, including the role of circadian rhythms and peripheral and central chemoreceptors to AD and heart disease; study the influences of developmental periods when remodeling occurs, and whether there are sex/gender differences in these pathways.
3. Prognosis and Treatment
  a. Evaluate whether SDB endotypes that reflect ANS function better predict HF and arrhythmias than global measures of SDB. Do cardiovascular outcomes differ in patients in whom AD itself plays a predominant mechanism for the sleep disorder and associated CVD; and/or in patients in whom AD responses to sleep disturbances or circadian misalignment mediate risk for heart disease.
  b. Investigate neuromodulation strategies that enhance upper airway muscle excitability and ameliorate sympathetic hyper-responsiveness based on activation of visceral afferent systems.
  c. Test, using both laboratory experimental studies and clinical trials, whether modulating carotid body and other sensory afferent output (via supplemental oxygen, neuromodulation, antioxidants, and or other interventions) stabilizes breathing and improves SNSA and secondarily, heart disease.

## Neurophysiological Mechanisms in Pulmonary Diseases and Interaction with Cardiac Function

### Background

Although the lungs and heart are separately innervated by afferent and efferent nerve pathways, there is a complex inter-relationship between these 2 organs in healthy and disease states that may be sensitive to intervention strategies.

**Figure 6:**
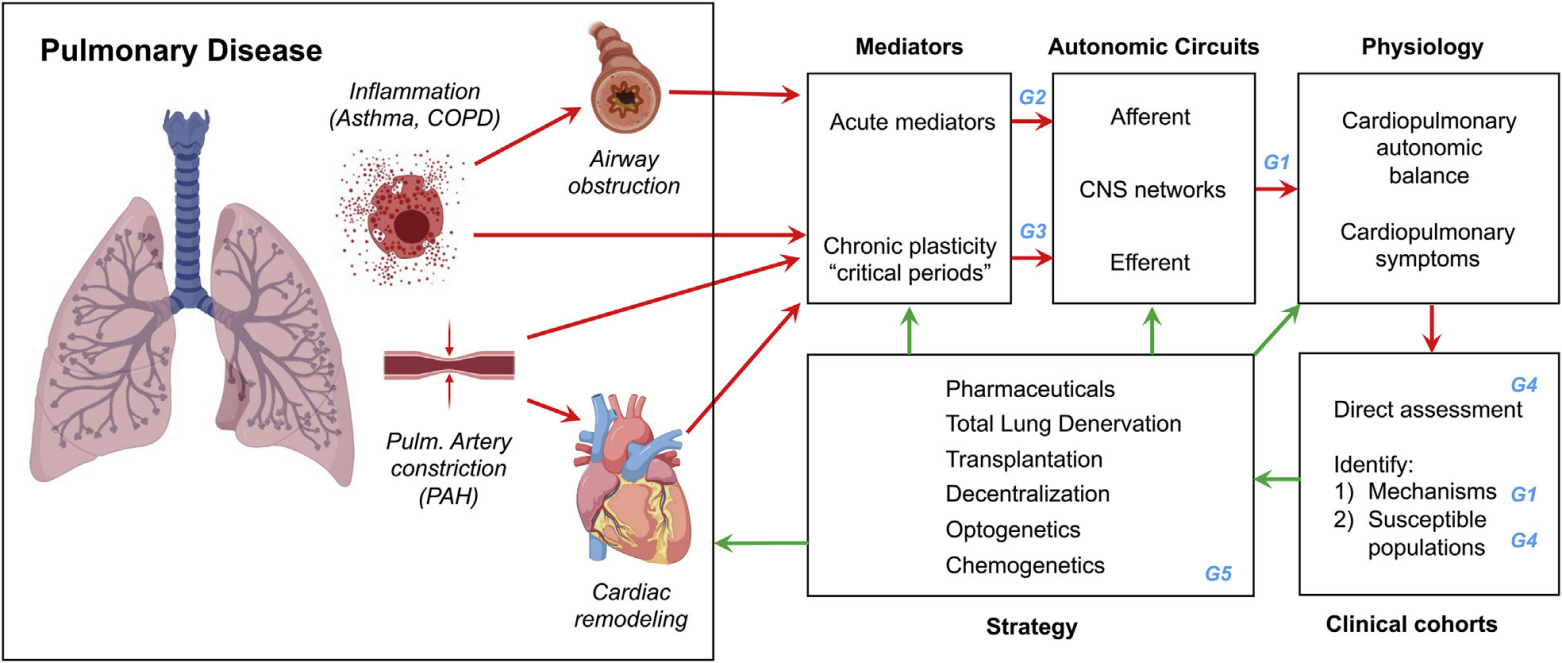
Neurophysiological Mechanisms in Pulmonary Disease and Interaction with Cardiac Function Schematic showing the knowledge gaps associated with the neurophysiological mechanisms in pulmonary diseases and their interaction with cardiac function. **Red arrows** denote physiological and pathophysiological interactions. **Green arrows** denote research implications. G labels **(blue)** denote the knowledge gaps identified in the main text. CNS = central nervous system; COPD = chronic obstructive pulmonary disease; PAH = pulmonary arterial hypertension.

In pulmonary arterial hypertension (PAH), autonomic dysfunction is secondary to right ventricular (RV) dysfunction, initiated by chronically elevated pulmonary artery pressures (Figure 6). The resultant sympathetic stimulation and parasympathetic withdrawal induces hypertrophy which initially enhances RV contractility. However, PAH is associated with long-term sympathetic overactivation (reduced HRV, impaired heart rate recovery, increased muscle SNA, and increased catecholamine plasma levels), which is linked with negative clinical outcomes (175). Furthermore, beta-adrenergic receptor density and protein kinase A (PKA)-mediated phosphorylation are reduced in the RV tissue of PAH patients with end-stage RV failure, and this results in increased passive stiffness of RV cardiomyocytes (176). Importantly, the efficacy of several neurotherapeutics have been shown in preclinical pulmonary hypertension models: beta-blocker therapy (bisoprolol, carvedilol, nebivolol), RAAS inhibitors (angiotensin receptor blockers, angiotensin-converting enzyme [ACE] inhibitors, recombinant ACE2), renal denervation, parasympathomimetics (pyridostigmine), and vagal nerve stimulation (177, 178, 179). Clinical translation of neurotherapeutics to treat PAH has been proven to be difficult thus far: recruitment of PAH patients is notoriously difficult (often single center), effective dosing is hampered due to side effects, and the demonstrated effects on RV function have been small.

Airway hyper-responsiveness is a hallmark of asthma (and some forms of chronic obstructive pulmonary disease [COPD]) that is defined as increased sensitivity to bronchospastic agents (Figure 6). Asthma medications including anti-inflammatory corticosteroids and newer biologic agents can substantively inhibit the inflammatory triggers of asthma, but they have little or no influence on reversing the hyper-reactive state (180,181). Previous studies have shown that cholinergic blockers evoke similar bronchodilation to beta adrenoceptor agonist in asthmatic subjects (182). This provides direct evidence that the majority of bronchospasm in asthma is parasympathetically induced (183, 184, 185). Recent findings suggest that airway hyper-responsiveness is dependent on intact vagus nerves (186), and that genetically removing only vagal C-fiber neurons abolished the hyper-reactivity (187), without altering the airway inflammation*.* As such, hyper-reactivity appears to be a function of vagal hyper-reflexivity.

Cardiac transplantation is associated with immediate cardiac decentralization, which includes loss of extrinsic efferent and afferent nerve connections, and an increased dependence on circulating catecholamines (Figure 6). Consequences include perturbations of resting cardiac physiology (increased heart rate, loss of HRV, increased nocturnal blood pressure) as well as exercise physiology (diminished maximal heart rate, slower heart rate increase and recovery, decreased contractility, decreased functional capacity) (188). Over the course of years after transplantation, there can be a process of reinnervation (which may improve functional capacity and outcomes) (189,190), but this is highly variable and not well-understood.

Pulmonary parasympathetic activity is enhanced in COPD and may play a critical role in the airway obstruction, airway hyper-responsiveness, and increased mucus production characteristic of this condition (184,191). Inhaled anticholinergic therapies have thus been the mainstay of COPD treatment (192,193). Similarly, disruption of pulmonary parasympathetic nerves in patients with COPD has the potential to provide long-lasting anticholinergic effects with reduction of symptoms and exacerbations. Targeted lung denervation (TLD) is a novel bronchoscopic therapy that disrupts the afferent and efferent pulmonary branches of the vagus nerve along the outside of the main stem bronchi using radiofrequency ablation (Figure 6). Initial studies have demonstrated the safety and feasibility of TLD and have shown trends toward improvements in symptoms and exacerbations (194,195); its efficacy is currently being assessed in the pivotal AIRFLOW-3 (Evaluation of the Safety and Efficacy of TLD in Patients With COPD) trial (196). Determining the appropriate radiofrequency ablation parameters in TLD is challenging because there are few direct assessments of afferent/efferent function. Because TLD also targets lung afferents, evaluation of cardiopulmonary reflexes such as respiratory sinus arrhythmia pre- and post-treatment may have the potential to assess the extent of pulmonary vagal denervation achieved with this approach (197,198).

### Knowledge gaps

1. There are critical gaps in our understanding of the contribution of specific afferent and efferent nerve subsets (199,200) to PAH, airway hyper-responsiveness, asthma, and COPD in clinical populations.
2. Little is known of the mechanisms by which local or systemic stimuli (including irritants, inflammation, and electrical) modulate afferent and efferent nerve activity, excitability, gene expression, and neuronal architecture.
3. Little is known of how chronic states, such as pulmonary disease or cardiopulmonary transplantation, alter afferent and efferent circuitry, plasticity, organ innervation, and reflex modalities. Evidence suggests that the ANS may be more sensitive during certain critical periods, but these and their importance have not been rigorously characterized.
4. A major challenge is identifying the patients with cardiopulmonary disease who are likely to be sensitive to autonomic modulation therapy. Such efforts are limited by poor direct assessment of autonomic function in patients, the possibility that cardiopulmonary disease states in clinical cohorts are dependent on a heterogeneous mix of autonomic and nonautonomic mechanisms, and the possibility that autonomic mechanisms are more prevalent at different stages of disease progression.
5. Despite the advances in cell-specific tools such as optogenetics and chemogenetics in animal models, there is a general lack of understanding how to modulate specific nerve subsets innervating specific organs in clinical populations.

### Research priorities

1. Continue to advance our basic understanding of the sensory/autonomic control of the heart and lungs by pursuing peripheral and chemical anatomical studies, biophysical electrophysiological analyses of the nerve sets involved, pharmacologic investigations regarding the neurotransmitters and inflammatory mediators involved in regulation of the nervous systems, and development of models using laboratory animals (mice, but also other species) that can provide insights into the role of sensory/autonomic nerves in cardiopulmonary diseases and following denervation.
2. Further develop tools for the direct assessment and modulation of afferent and efferent nerve innervation and activity in clinical populations.
3. Determine the contribution of afferent and efferent nerve activity and autonomic reflexes to chronic states, such as pulmonary disease (eg, PAH, asthma, and COPD) or cardiopulmonary transplantation.

## Summary

The NHLBI workshop participants identified multiple themes across our 6 foci to summarize the current state of knowledge in the basic and clinical sciences related to neural control of cardiac, sleep, and circadian rhythm, and lung physiology. Advances in ANS research in recent years have led to numerous discoveries that have initiated a return of this classic discipline back to the center stage of health and disease. At the same time, a number of challenges and knowledge gaps must be addressed to clarify the role of ANS in cardiopulmonary diseases and sleep and circadian rhythm dysfunction, and to harness the power of ANS science for human therapy. Important gaps in knowledge were identified as were proposed novel approaches to advance basic, translational and population sciences.

The critical need to determine fundamental mechanisms of neural control of the nerve–organ interface (heart and lung) to identify key regulatory circuits that can be targeted for therapies was a unanimous informed opinion. Overarching themes were identified. For instance, several fundamental basic science questions remain unanswered regarding the mechanisms by which nerves control cardiopulmonary physiology. The ANS profoundly influences cardiopulmonary regulation with neural circuits in millisecond timescales. These neural circuits transduce pathophysiological signals and the neural structures themselves undergo neural remodeling. Neuroinflammation is now recognized as a key pathophysiological change. Specific areas of focus should include the importance of normal integrated cardiopulmonary control and the potential pathological impact of altered neural signaling the cellular, tissue, organ, and whole individual levels. Multiple challenges to exploring these questions relate to lack of roadmaps, limitations of tools and technologies for both animal and cellular models and limited mechanistic studies in humans to inform the design of clinical trials. To overcome these research challenges, greater emphasis on experimental rigor and methodological transparency is needed to control for and assess the influence of neural signaling in cardiopulmonary physiology.

Many published animal studies have not rigorously accounted for the complexity of neural circuit readouts, and additionally, sex as a biological variable has been inadequately studied. Human studies often do not report the sleep–wake cycle and do not consider circadian rhythm influences nor account for environmental factors responsible for circadian entrainment. Human studies of neural control of cardiopulmonary function are limited by the absence of reliable biomarkers of neural remodeling, and recent studies in this area provide encouragement to define disease progression and a way to “dose” neuromodulation therapies. A human-to–cell system approach is appealing in terms of deep phenotyping of human autonomic tissue, recapitulating autonomic cells from patients with dysautonomia (human induced pluripotent stem cell technology), follow-up phenotyping of patients after denervation procedures (eg, heart–lung transplant patients) or implanted stimulators, and clarifying ANS endophenotypes that derive therapeutic utility. It is worthwhile to mention that although not a focus of this workshop, there are key ANS-specific opportunities to consider pertaining to COVID-19 and post-acute sequelae of COVID-19, a situation in which physiology, neuroscience, and immunology converge, and ACE2 biology is implicated.

Human mechanistic studies of neural control that are informed by appropriate large animal models have the highest translational value for advancing human physiology and pathophysiology. Greater knowledge of neural control–related variables in populations and how these exert an impact, or are affected by, disease states that lead to arrhythmias, HF, and lung diseases has high potential to enable development of specific interventions and most importantly novel preventive strategies. Risk stratification for vulnerability to SCD can lead to pre-emptive strategies for drug administration and delivery to ameliorate disease progression and reduce risk. Elucidating age-, sex-, and race-specific aspects of ANS pathophysiology in cardiopulmonary disease and pathophysiology of sleep/circadian dysfunction will inform personalized risk stratification and interventional strategies. Finally, new therapeutic strategies may also blunt the risk of adverse cardiovascular consequences in populations that experience persistent disruptions neural control (eg, sleep and circadian rhythm disorders).

The substantial and ongoing contributions of the National Institutes of Health Common Fund’s SPARC program to the understanding of the neural control of cardiac and pulmonary physiology were apparent at this workshop. SPARC-supported efforts, including those by many participants, have generated data using modern neuroanatomical techniques and electrical stimulation coupled with cutting-edge biosensors to describe and probe comprehensively the neural circuits underlying cardiopulmonary function. The program has also led the way in promoting data sharing with annotation for semantic interoperability. As a result, an ever-increasing corpus of this SPARC-generated data is available, documented, annotated, and citable on the SPARC Portal. Indeed, 1 theme emerging from this workshop summary is the importance of comparing and linking findings from different model systems and disease states. This can be difficult when investigators only have access to top-line conclusions and narrative interpretations, rather than the underlying analysis. The SPARC Portal resources are designed to address this need. Both newly interested and seasoned investigators can visit the portal to find data sets suitable for joining to their own, or for launching new computational analyses and predictive simulations. Such efforts could address the knowledge gaps identified here, advancing a new generation of therapeutic neuromodulation devices and protocols for cardiopulmonary applications.

Overall, the workshop participants are hopeful that this overview of the current state of autonomic control in cardiopulmonary disease and sleep and circadian dysfunction, which focused on existing knowledge gaps and identification of key research priorities and resources, such as SPARC, will stimulate investigative initiatives that will address critical areas and contribute to pivotal advances in the basic and clinical sciences related to neural control of cardiac, sleep/circadian, and lung physiology.

## Funding Support and Author Disclosures

This workshop was supported by the National Heart, Lung, and Blood Institute (NHLBI) and the National Institutes of Health (NIH) Office of the Director. Dr Ajijola is supported by NIH grants DP2 OD024323, OT2 OD028201, and OT2 OD023848, Dr Chen was supported by NIH grants OT2 OD028190, R01 HL139829, and the Burns & Allen Chair in Cardiology Research, Cedars-Sinai Medical Center. Dr Clancy has received research funding from NIH grants OT2 OD026580, OT2 OD026580, R01 HL152681, R01 HL128170, and U01 HL126273, InCarda Therapeutics, and the Department of Physiology and Membrane Biology Research Partnership Fund, Oracle cloud for research allocation. Dr Delisle is supported by NIH grants R01 HL153042, R01 HL141343, and the American Heart Association grant 20IPA35320141. Dr Habecker was supported by NIH grants R01 HL093056 and R01 HL146822. Dr Handoko has received an educational grant from Novartis and Boehringer Ingelheim. Dr Redline has received research funding from NIH grants R35 HL135818, R01 HL133684, U10 HD036801, R01 HL137234, R01 DK118736, R01 AG056331, and U24 HL140412, and Jazz Pharmaceuticals. Dr Ripplinger is supported in part by research funding from NIH grants OT2 OD026580, R01 HL093056, and R01 HL111600. Dr Somers is supported by NIH grants R01 HL065176 and R01 HL134885. Dr Stavrakis was supported by NIH grant R21 AG057879. Dr Taylor-Clark is supported by NIH grants R01 HL152219, U01 NS113868, OT2 OD023854, R21 DK124894, R01 DK110366, and U01 DK110366. Dr Undem is supported by NIH grant R35 HL155671. Dr Zucker has received support from Sorrento Therapeutics, Inc. and from NIH grant R01 HL126796. Dr Shivkumar is supported by NIH grants OT2 OD028201 and OT2 OD023848. Dr Ajijola is a cofounder and equity holder of NeuCures; has served as a consultant for Merck and Biosense-Webster Inc. Dr Gold has received clinical trial research funding from Boston Scientific and Medtronic; and has been a consultant for Boston Scientific, CVRx. and Medtronic. Dr Habecker is a co-inventor of a technology (intracellular sigma peptide) that Oregon Health and Science University has licensed to NervGen Pharma Corp. Dr Handoko has received research funding from Vifor Pharma; and has been a consultant for Novartis, Boehringer Ingelheim, Vifor Pharma, AstraZeneca, Bayer, MSD, Daiichi Sankyo, and Quin. Dr Hummel has been a scientific advisory board member; and holds equity in Nuvaira, Inc. Dr Redline has received consulting fees from Jazz Pharmaceuticals and Apnimed Inc. Dr Redline has received research funding from Jazz Pharmaceuticals. Dr Somers has served as a consultant for Bayer, Respicardia, Baker Tilly, and Jazz Pharmaceuticals; and has served on the scientific advisory board of the Sleep Number Corporation. Dr Shivkumar is a cofounder and equity holder of NeuCures Inc. Dr Simon is supported in part by NIH grant R01 AG058659, serves on a clinical trial steering committee for Janssen, and has consulted for Acceleron and Bial. Dr Mehra is supported by NIH grants U01HL125177, UH3HL140144 and the American Heart Association AHA 18SFRN34170013. Dr Arora is supported by NIH grants R01 HL140061, R01 HL125881, Technology Development Program, NIH Center for Accelerated Innovations at Cleveland Clinic, the American Heart Association Strategically Focused Research Networks Atrial Fibrillation Center, and has ownership interest in Rhythm Therapeutics, Inc. Dr Handoko is supported by the Dutch Heart Foundation (Senior Clinical Scientist grant 2020T058); has received research funding from Vifor Pharma; and has been a consultant for Novartis, Boehringer Ingelheim, Vifor Pharma, AstraZeneca, Bayer, MSD, Daiichi Sankyo, and Quin. The views expressed in this article are those of the authors and do not necessarily represent the views of the National Heart, Lung, and Blood Institute; the National Institutes of Health; or the U.S. Department of Health and Human Services. All other authors have reported that they have no relationships relevant to the contents of this paper to disclose.
